# Dinickel–Salphen Complexes as Binders of Human Telomeric Dimeric G‐Quadruplexes

**DOI:** 10.1002/chem.201700276

**Published:** 2017-03-24

**Authors:** Chun‐Qiong Zhou, Ting‐Cong Liao, Zi‐Qi Li, Jorge Gonzalez‐Garcia, Matthew Reynolds, Min Zou, Ramon Vilar

**Affiliations:** ^1^ Department of Chemistry Imperial College London, South Kensington London SW72AZ UK; ^2^ Guangdong Provincial Key Laboratory of New Drug Screening School of Pharmaceutical Sciences Southern Medical University Guangzhou 510515 P. R. China

**Keywords:** dimeric quadruplex DNA, DNA recognition, metal salphen, telomere⋅nickel

## Abstract

Three new polyether‐tethered dinickel–salphen complexes (**2 a**–**c**) have been synthesized and fully characterized by NMR spectroscopy, mass spectrometry, and elemental analyses. The binding affinity and selectivity of these complexes and of the parent mono‐nickel complex (**1**) towards dimeric quadruplex DNA have been determined by UV/Vis titrations, fluorescence spectroscopy, CD spectroscopy, and electrophoresis. These studies have shown that the dinickel–salphen complex with the longest polyether linker (**2 c**) has higher binding affinity and selectivity towards dimeric quadruplexes (over monomeric quadruplexes) than the dinickel–salphen complexes with the shorter polyether linkers (**2 a** and **2 b**). Complex **2 c** also has higher selectivity towards human telomeric dimeric quadruplexes with one TTA linker than the monometallic complex **1**. Based on the spectroscopic data, a possible binding mode between complex **2 c** and the dimeric G‐quadruplex DNA under study is proposed.

## Introduction

**Figure 1:**
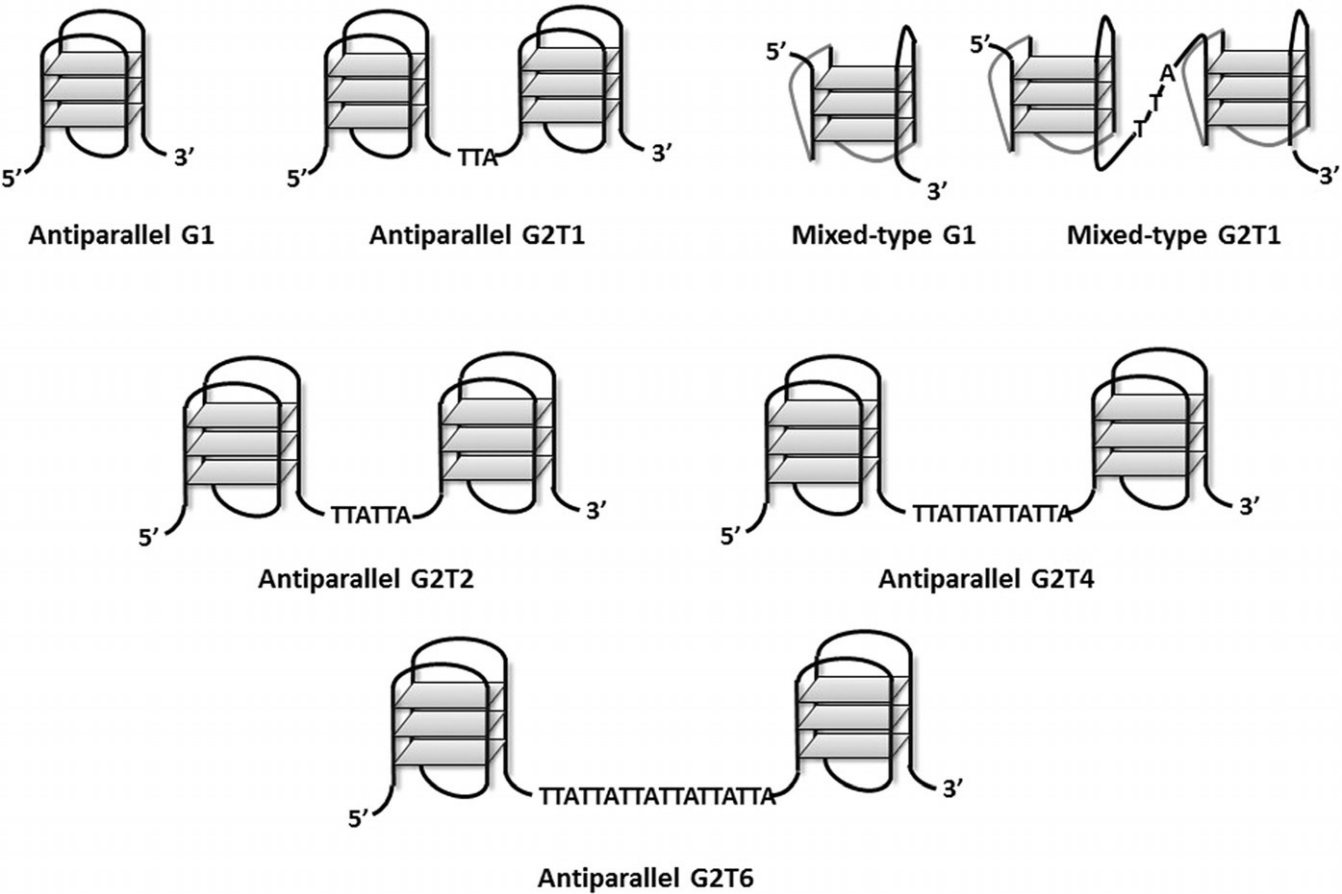
Schematic representation of various monomeric and dimeric G‐quadruplexes.

Human telomeric DNA is composed of hundreds of 5′‐TTAGGG repeats that end in a single‐stranded overhang of around 200 nucleobases. Under physiological conditions, this sequence can fold into a tetra‐stranded helical arrangement known as G‐quadruplex DNA. This structure has attracted significant attention due to its proposed role in telomere maintenance and consequently its potential as a target for the development of new anticancer therapies.^1^, ^2^, ^3^, ^4^ Therefore, a large number of small molecules have been developed over the past two decades with the aim of selectively binding and stabilizing G‐quadruplex DNA.^5^, ^6^, ^7^, ^8^, ^9^ Although the structures of single G‐quadruplexes containing four repeats of the 5′‐TTAGGG sequence have been studied in detail,^10^, ^11^, ^12^, ^13^, ^14^, ^15^ less is known about the higher‐order structures formed by longer telomeric sequences.^16^, ^17^, ^18^, ^19^ The latter structures, though difficult to study in vitro, are likely to be physiologically more relevant because the single‐stranded overhang of telomeric DNA can potentially fold into oligomers containing as many as ten consecutive G‐quadruplexes linked by TTA spacers (see Figure 1). In addition to the telomere, multimeric G‐quadruplexes have also been proposed to form in other oligonucleotide sequences. For example, r(GGGGCC)_*n*_ repeats can lead to the formation of multimolecular G‐quadruplex RNA structures, which have been proposed to be relevant in amyotrophic lateral sclerosis (ALS).^20^, ^21^, ^22^, ^23^

Though a large number of small molecules have been previously developed as single G‐quadruplex DNA binders,^5^, ^6^, ^7^, ^8^, ^9^ comparatively very few have been studied (or indeed specifically designed) as binders for multimeric G‐quadruplex structures.^24^, ^25^, ^26^, ^27^, ^28^, ^29^, ^30^, ^31^, ^32^ This includes a chiral cyclic helicene proposed to bind in the cleft between two human telomeric G‐quadruplexes linked by a TTA spacer.^25^ Other examples are oxazole‐based “click” ligands that stabilize tandem parallel‐folded G‐quadruplex motifs^29^ and a chiral supramolecular dinickel(II) complex with selectivity for higher‐order telomeric DNA G‐quadruplexes.^24^ Tetraphenylethene (TPE) derivatives have also been shown to bind to G‐quadruplex multimers and their selectivity tuned by changing the substituents^30^ around the aromatic core. More recently, polyether‐linked di‐berberines have been reported to have high selectivity for *anti*‐parallel dimeric G‐quadruplex DNA.^32^

**Scheme 1:**
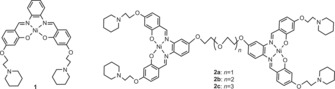
Structures of nickel–salphen complexes **1** and **2 a**–**c**.

Over ten years ago, we reported the first example of a G‐quadruplex binder based on metal salphens.^33^ This was followed by several other reports,^34^, ^35^, ^36^, ^37^, ^38^, ^39^, ^40^ which include the structural characterization of nickel(II)‐ and copper(II)–salphen complexes bound to a G‐quadruplex from a human telomeric sequence,^35^ the use of platinum(II) salphens as luminescent probes for G‐quadruplexes,^36^, ^39^ and the demonstration that these complexes can also inhibit telomerase.^35^, ^37^, ^38^, ^39^ Because this family of compounds displayed high binding affinities to human telomeric G‐quadruplex DNA, we were interested in establishing their affinity and selectivity for multimeric G‐quadruplex structures. Here, we report on the synthesis of three new dinickel–salphen complexes (**2 a**–**c**) with different‐length polyether linkers (Scheme 1) and on their affinity and selectivity towards dimeric quadruplex DNA. For comparison, we have also studied the affinity towards dimeric G‐quadruplexes of the previously reported mono‐nickel(II)–salphen complex **1**.

## Results and Discussion

### Synthesis of dinickel–salphen complexes

**Scheme 2:**
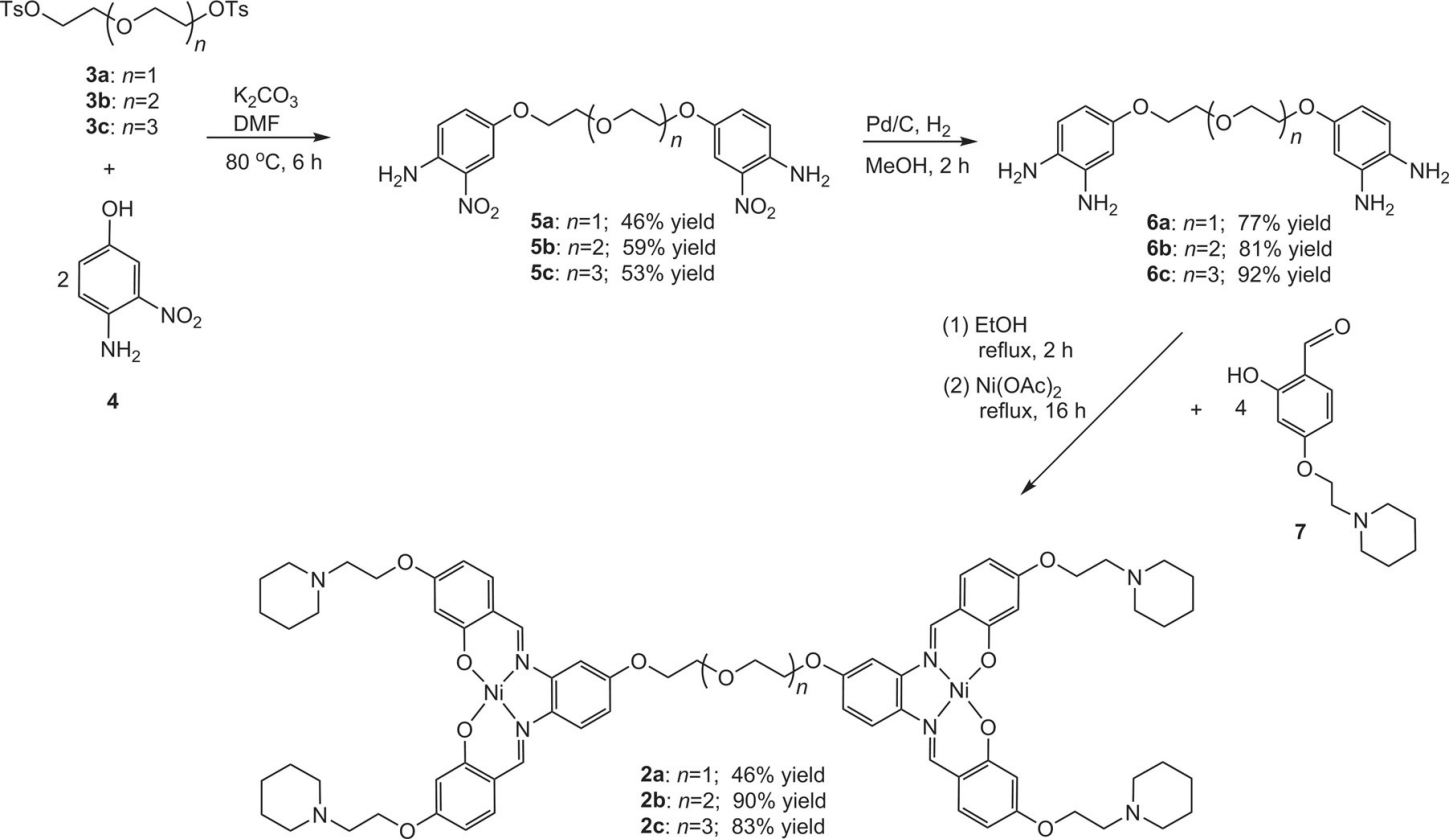
Synthetic route for the preparation of dinickel–salphen complexes **2 a**–**c**.

The dinickel–salphen complexes **2 a**–**c** were synthesized as outlined in Scheme 2. Compounds **3 a**–**c** were reacted with 4‐amino‐3‐nitrophenol (**4**) in dimethylformamide at 80 °C to yield compounds **5 a**–**c**, followed by reduction of the nitro group to give the tetra‐amine compounds **6 a**–**c**. These compounds were reacted with four equivalents of the piperidine‐substituted aldehyde **7** in ethanol at reflux for 2 h. To this reaction mixture, two equivalents of Ni(OAc)_2_⋅4 H_2_O were added to yield the final complexes **2 a**–**c** after 16 h of reflux. Compounds **2 a**–**c**, **5 a**–**c**, and **6 a**–**c** were fully characterized on the basis of NMR spectroscopy (^1^H and ^13^C), mass spectrometry (LR and HR), and elemental analysis (see the Experimental Section). Compounds **3 a**–**c** and **7** were prepared according to reported protocols.^32^, ^33^, ^36^, ^41^

### UV/Vis titration to determine DNA affinity

**Figure 2:**
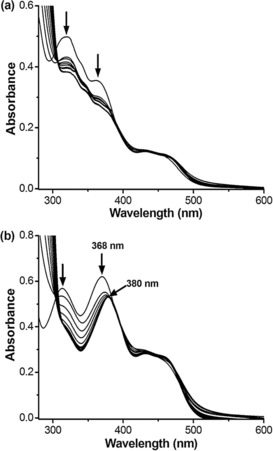
Representative examples of UV/Vis titration of 20 μm dinickel complexes **2 a** (a) and **2 c** (b) with increasing concentration (from 0 to 10 μm in 10 mm Tris‐HCl and 100 mm NaCl, pH 7.04) of G2T1 DNA.

The DNA binding affinities of complexes **2 a**–**c** and **1** towards the K^+^ stabilized mixed‐type monomeric quadruplex G1 and dimeric quadruplex G2T1, and the Na^+^ stabilized antiparallel G1 and G2T1 (see Figure 1 and Table 3), were determined by UV/Vis titrations. The UV/Vis spectra of these nickel(II) complexes showed similar patterns, with two strong absorption bands in the region 310–330 nm (associated with intraligand π–π^*^ transitions) and in the region 360–390 nm (which involves both the ligand and the metal center).^35^ Addition of increasing amounts of G2T1 to these complexes resulted in considerable hypochromicity (15–34 %) for the two peaks at 310–330 nm and 360–390 nm (Figure 2, plus Figures S28 and S29 in the Supporting Information). Interestingly, the addition of G2T1 resulted in a noticeable redshift of complex **2 c** (12 nm in 100 mm NaCl buffer and 7 nm in 100 mm KCl buffer; Figure 2 b and Figure S28c) and complex **1** (16 nm in 100 mm NaCl buffer and 13 nm in 100 mm KCl buffer; Figures S28d and S29d). These spectral features are indicative of an end‐stacking binding mode rather than groove binding.^35^ On the other hand, upon addition of increasing amounts of G2T1, the redshifts of complexes **2 a** and **2 b** were considerably smaller (under 4 nm; see Figure 2 a, Figures S28 and S29), suggesting that the interaction of these complexes with DNA through end‐stacking is relatively weak. For comparison, the interaction between these nickel(II) complexes and CT DNA was also studied (see Figure S32 in the Supporting Information). Upon addition of increasing amounts of CT DNA to the corresponding compound, hypochromicity was observed (between 40 and 57 %) but no redshift, suggesting that these complexes are not good duplex DNA intercalators but may possibly act as duplex DNA groove binders.

The intrinsic binding constants of the four complexes towards G2T1, G1, and CT DNA were determined by monitoring the changes of the absorption with the increase of DNA concentration and the results are summarized in Table 1 and Table S1. The di‐nickel complexes **2 a**–**c** have slightly lower apparent binding constants (*K*_a_ values) for the monomeric antiparallel G1 structure than the mono‐nickel parent complex **1**. On the other hand, complex **2 c** showed the highest binding affinity towards the dimeric antiparallel and mixed‐type G2T1 structures, followed closely by complex **1**. Interestingly, **2 c** also displayed the best selectivity for antiparallel G2T1 versus G1 and CT‐DNA (30‐fold and 297‐fold, respectively, Table 1), whereas the selectivity of complex **2 c** for mixed‐type G2T1 versus G1 is only six‐fold (Table S1 in the Supporting Information).

**Table 1:** Apparent binding constants (*K*_a_ values, M^−1^) of complexes **2 a**–**c** and **1** for G2T1, G1, and CT DNA in 10 mm Tris‐HCl and 100 mm NaCl (pH 7.04) by UV/Vis spectroscopy.

Complex	*K*_a_ (G2T1)	*K*_a_ (G1)	*K*_a_ (CT DNA)	Selectivity for G2T1 vs. G1	Selectivity for G2T1 vs. CT‐DNA
**2 a**	^[a]^1.08±0.24×10^6^	^[b]^1.27±0.24×10^6^	2.54±0.24×10^5^	1	4
**2 b**	^[a]^2.06±0.40×10^6^	^[a]^8.30±0.75×10^5^	2.05±0.08×10^5^	3	10
**2 c**	^[b]^3.15±0.42×10^7^	^[b]^1.05±0.20×10^6^	1.06±0.11×10^5^	30	297
**1**	^[a]^2.34±0.32×10^7^	^[b]^4.62±0.64×10^6^	1.20±0.15×10^5^	5	195

[a] Absorption measured at 310 nm; [b] absorption measured at 370 nm.

### Circular dichroism spectroscopic studies

**Figure 3:**
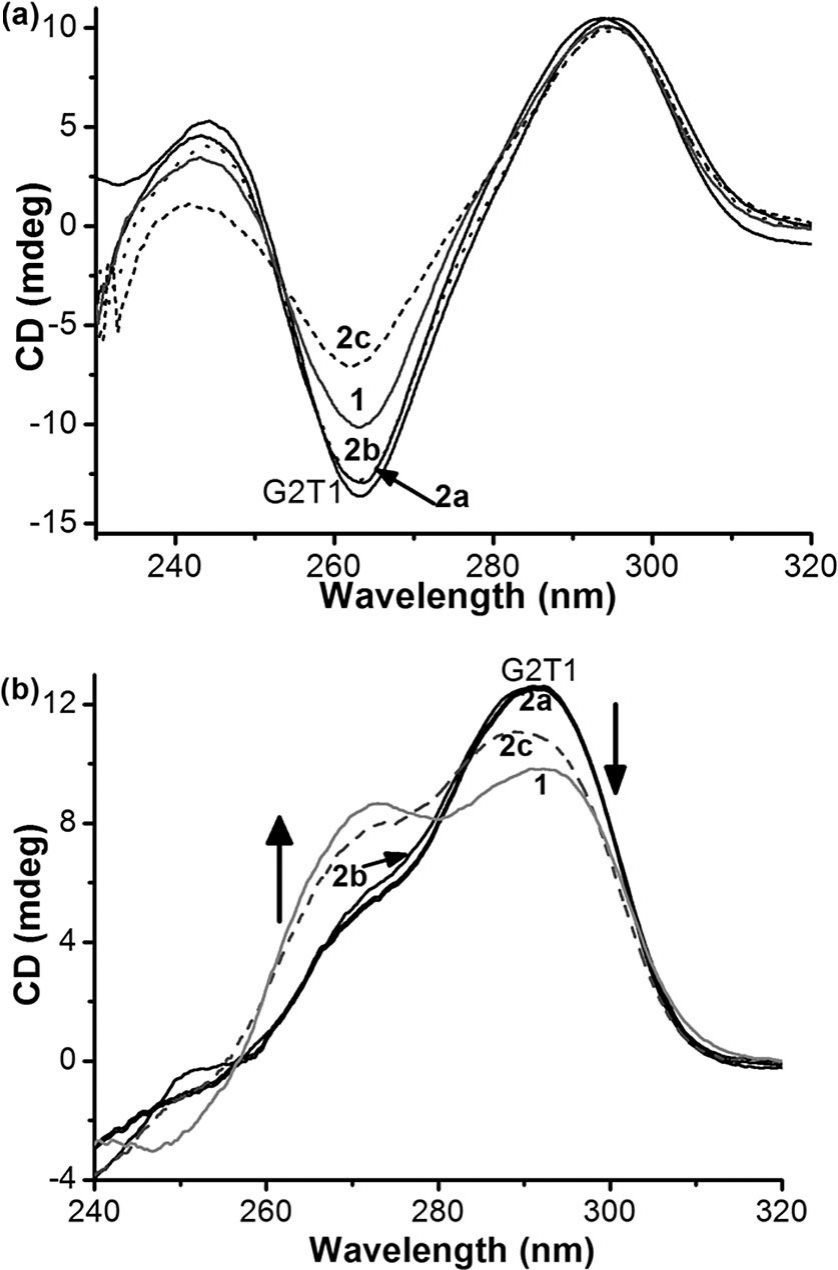
CD spectra of G2T1 (2.5 μm) with or without complexes **2 a**–**c** (5 μm) and **1** (10 μm) in 10 mm Tris‐HCl (pH 7.04) and (a) 100 mm NaCl, and (b) 100 mm KCl.

Having established that the nickel(II) complexes bind to the dimeric G2T1 DNA structures, we were interested in studying the effect of the binding on the structure of the G‐quadruplexes. Therefore the interactions of complexes **2 a**–**c** and **1** with G2T1 were investigated by CD spectroscopy (Figure 3). We first investigated the Na^+^ stabilized antiparallel dimeric quadruplex G2T1. Upon addition of **2 a** and **2 b**, no significant changes in the ellipticity of G2T1 were observed, while addition of **1** and **2 c** induced minor changes in the negative ellipticity at 265 nm (Figure 3 a). These results suggest that the complexes do not bring about major structural changes in the antiparallel conformation of the G2T1 quadruplex structure.^42^ We then investigated the K^+^ stabilized mixed‐type quadruplex structure. Though no significant changes were observed in the presence of complexes **2 a** and **2 b**, the addition of **1** and **2 c** caused a marked increase of the intensity of the positive peak at approximately 265 nm (associated with the parallel conformation), and a decreased intensity of the positive peak at approximately 295 nm (associated with the antiparallel conformation). These results suggest that complexes **2 c** and **1** promote the formation of parallel quadruplex in K^+^ buffer.^30^, ^34^, ^43^

**Figure 4:**
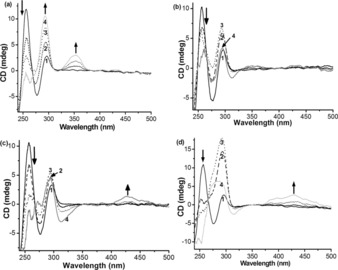
CD spectra of nonannealed G2T1 (2.5 μm) in 10 mm Tris‐HCl (pH 7.04) in the presence of (a) **2 a**, (b) **2 b**, (c) **2 c**, and (d) **1**: (1) 0 equiv; (2) 1 equiv; (3) 2 equiv; (4) 4 equiv.

CD spectroscopy was also used to determine the potential templating effects of these nickel complexes on the formation of G2T1 quadruplex DNA. Non‐annealed G2T1 in the absence of K^+^ or Na^+^ and without added metal complex, showed the characteristic positive ellipticity at ca. 250 nm consistent with a single‐stranded DNA sequence (Figure 4). Upon addition of each of the four nickel complexes under study, the signal centered at 250 nm decreased while the signals associated to the formation of quadruplex DNA increased. Interestingly, the three di‐nickel complexes induced mainly the formation of an antiparallel quadruplex structure (with positive ellipticity centered at ca. 295 nm, Figure 4 a–d). Though this is also the case for compound **1** at low concentrations, upon increasing the amount of compound added a positive shoulder peak at 265 nm appeared, which suggests the formation of mixed‐type quadruplex DNA (Figure 4 d).^30^, ^34^, ^35^, ^43^ For complexes **2 b**, **2 c**, and **1** we noted a decrease in the overall intensity of the CD spectra at the highest compound concentrations used, which might be due to aggregation/precipitation of DNA induced by the compounds.^35^

Dinickel complexes **2 a** and **2 c** displayed induced CD signals in the presence of G2T1 quadruplex DNA: at 352 nm (with positive ellipticity) for the former, and at 311 nm (with negative ellipticity) and 431 nm (with positive ellipticity) for the latter (Figure 4 a and c). Complex **1** showed a broad induced CD signal with positive ellipticity at 431 nm (Figure 4 d). No significant induced CD signals were observed for **2 b**.

**Figure 5:**
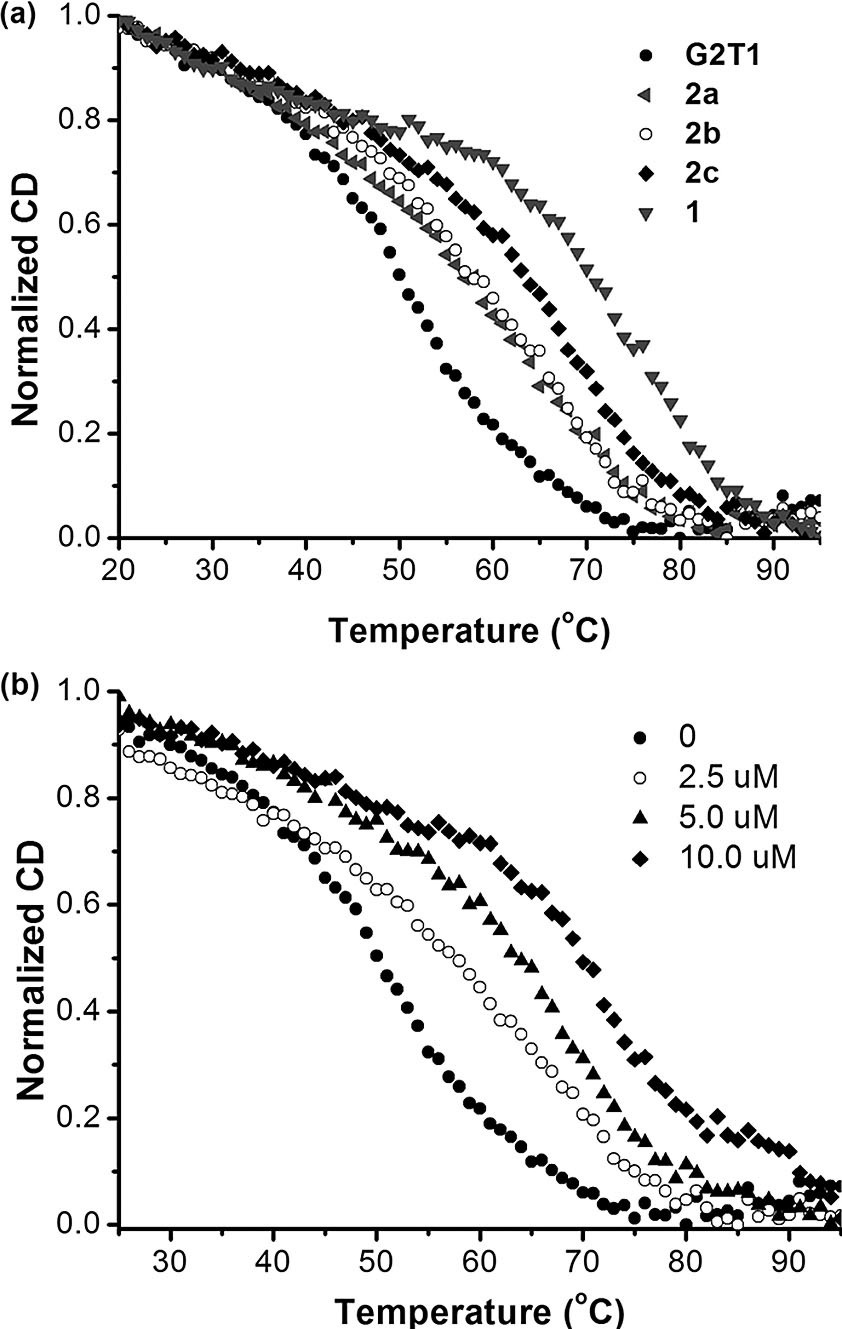
CD‐melting profiles at 295 nm for G2T1 (2.5 μm) in 10 mm Tris‐HCl and 100 mm NaCl (pH 7.04) in the presences of: (a) complexes **2 a**–**c** (5.0 μm) and **1** (10.0 μm); and (b) increasing concentrations of complex **2 c** (0, 2.5, 5.0, and 10.0 μm).

CD melting assays were then used to further assess the affinity and thermal stabilization of the nickel(II) complexes towards dimeric G‐quadruplex G2T1. These experiments were carried out in 10 mm Tris‐HCl and 100 mm NaCl buffer to ensure that the G‐quadruplex was present in a single antiparallel conformation (rather than mixed conformations as in the case with K^+^; see Figures S33 and S34 in the Supporting Information). Upon increasing the temperature, the signals at 295 nm with positive ellipticity and at 260 nm with negative ellipticity (characteristic of antiparallel conformation), decreased until their disappearance when the G‐quadruplex was completely unfolded (Figure S34 b–e). The melting of G2T1 was then carried out in the presence of the different nickel(II) complexes and the results are summarized in Figure 5 a. Complexes **2 a** and **2 b** (at a 2:1 molar ratio between complex and G2T1) displayed relatively low Δ*T*_m_ values: 7.7 and 8.4 °C, respectively. On the other hand, the dinickel complex **2 c** and mono‐nickel complex **1** displayed significantly higher Δ*T*_m_ values (Figure 5 a): 14.1 °C for complex **2 c** (2:1 complex‐to‐G2T1 ratio) and 20.8 °C for complex **1** (4:1 complex‐to‐G2T1 ratio). For complex **2 c**, we also investigated changes in Δ*T*_m_ upon increasing the concentration of the complex; as can be seen in Figure 5 b, a significant thermal stabilization (Δ*T*_m_=19.8 °C) was observed at a 4:1 ratio of **2 c** to G2T1.

These results suggested that the dinickel complex **2 c** (with the longest polyether linker) and monomeric nickel complex **1** showed the higher binding affinities and thermal stabilization toward G2T1 as compared to the dinickel complexes **2 a** and **2 b** (with the shorter polyether linkers), which is consistent with the results obtained through UV/Vis titrations. Furthermore, it should also be noted that complexes **2 c** and **1** exhibited higher than or comparable affinities/thermal stabilization than other G2T1 binders previously reported in the literature (Table S2 in the Supporting Information).^24^, ^26^, ^27^, ^29^, ^30^, ^31^

We then investigated the binding of **1** and **2 c** towards dimeric quadruplexes linked by one, two, four, or six TTA subunits named G2T1, G2T2, G2T4, and G2T6, respectively (see Figure 1 for schematic representation of these structures and Table 3 for sequences). The Δ*T*_m_ values (Table 2 and Figure S35 for their CD spectra) of these dimeric G‐quadruplexes upon addition of complex **2 c** decreased with the length of the TTA linkers, indicating that this complex has higher affinity for dimers with short TTA linkers. In contrast, the Δ*T*_m_ values of the different dimeric G‐quadruplexes in the presence of the mono‐nickel complex **1** showed little change regardless of the length of the TTA linkers (Table 2). These results indicate that, although complex **1** has higher binding affinities and induces higher thermal stabilizations for both monomeric and dimeric G‐quadruplexes, the dinickel complex **2 c** has better selectivity: seven‐fold higher preference for the dimeric G‐quadruplex G2T1 than for the monomeric G1 (whereas the selectivity of complex **1** for G2T1 vs. G1 is only two‐fold).

**Table 2:** Quadruplex DNA stability measurements from CD‐melting curves with complexes **2 c** and **1** in 10 mm Tris‐HCl and 100 mm NaCl (pH 7.04). The amount of complex added was such that all samples had a 2:1 ratio of nickel–salphen with respect to each G‐quadruplex unit (e.g., [**1**]:[G2T1]=4:1; [**1**]:[G1]=2:1; [**2 c**]:[G2T1]=2:1; [**2 c**]:[G1]=1:1).

Complex	**Δ** *T*_m_ [°C]				
	G2T1	G2T2	G2T4	G2T6	G1
**1**	20.8	25.1	25.5	23.5	13.4
**2 c**	14.1	11.9	8.1	6.3	2.0

### Native gel electrophoresis

**Figure 6:**
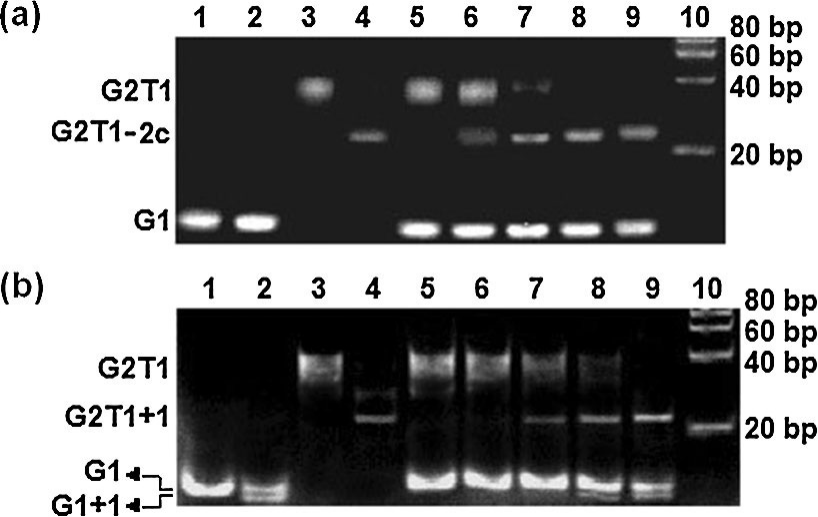
Native gel electrophoretic analysis of G1, G2T1, and their mixture in the presence of complex **2 c** (a) and complex **1** (b) in 10 mm Tris‐HCl and 100 mm NaCl (pH 7.04). (a) Lanes 1 and 2: G1 (16 μm) in the absence and presence of complex **2 c** (16 μm); lanes 3 and 4: G2T1 (8 μm) in the absence and presence of complex **2 c** (16 μm); lane 5: a mixture of G1 (16 μm) and G2T1 (8 μm); lanes 6–9: mixtures of G1 (16 μm) and G2T1 (8 μm) in the presence of 4, 8, 16, and 32 μm of complex **2 c**, respectively; lane 10: DNA ladder. (b) Lanes 1 and 2: G1 (16 μm) in the absence and presence of complex **1** (32 μm); lanes 3 and 4: G2T1 (8 μm) in the absence and presence of complex **1** (32 μm); lane 5: a mixture of G1 (16 μm) and G2T1 (8 μm); lanes 6–9: mixtures of G1 (16 μm) and G2T1 (8 μm) in the presence of 8, 16, 32, and 64 μm of complex **1**, respectively; lane 10: DNA ladder.

Based on previously reported protocols,^24^, ^30^, ^32^ the selectivity of complex **2 c** for G2T1 over G1 was investigated by gel electrophoresis (Figure 6 and Figures S37 and S38 in the Supporting Information). The gel shown in Figure 6 a indicates that addition of **2 c** to antiparallel G1 (in the presence of Na^+^) did not lead to the appearance of any new band (lane 2), suggesting that this compound does not form a stable complex with monomeric G1 under the gel electrophoresis conditions. By contrast, the presence of complex **2 c** increased the mobility rate of the antiparallel dimeric quadruplex G2T1, which could be rationalized by the formation of a more compact G2T1 upon interaction with **2 c** (lane 4), as has been previously proposed for other G‐quadruplex binders.^30^ To further verify the preference of **2 c** for G2T1 over G1, the complex was incubated with a mixture of G1 and G2T1 and the mixture analyzed by gel electrophoresis. As a control, a mixture of G1 and G2T1 in the absence of **2 c** was also analyzed by gel electrophoresis; as can be seen in Figure 6 (lane 5), this sample gave the characteristic bands corresponding to intramolecular monomeric (G1) and dimeric (G2T1) G‐quadruplexes. After addition of complex **2 c** to the G1 and G2T1 mixture, a new band corresponding to complex G2T1‐**2 c** appeared. This band became more intense upon addition of increasing amounts of **2 c** to the G1/G2T1 mixture, but no changes were observed for the band associated with G1 (lanes 6 to 9 in Figure 6 a).

An analogous experiment was carried out with the mono‐nickel complex **1** (Figure 6 b). The presence of **1** increased the mobility rate of the dimeric quadruplex G2T1, which is analogous to what we observed with **2 c**. Interestingly, we also observed a new band for the monomeric G1 structure upon addition of complex **1**, suggesting that this compound does not discriminate between the monomeric and dimeric G‐quadruplex structures (see Figure 6 b, lanes 2 and 4). This was further confirmed upon addition of complex **1** to a mixture containing G1 and G2T1: two new bands corresponding to complexes G2T1‐**1** and G1‐**1** were present (lanes 7–9).

The same set of gel electrophoresis experiments were carried out with the K^+^ stabilized parallel/antiparallel mixed‐type G2T1 and G1 structures (Figure S38). The behaviors for both **2 c** and **1** are analogous to what was observed with the Na^+^ stabilized parallel G2T1 and G1 structures, namely **2 c** has higher binding selectivity for G2T1 versus G1 than complex **1**. An interesting observation was that, when the molar ratio of complex **2 c** and G2T1 reached 4:1, the whole G2T1 structure could not be converted into complex G2T1‐**2 c** (Figure S38a, lane 8). However, as described above, all the antiparallel G2T1 structure could be converted into complex G2T1‐**2 c** when the molar ratio of complex **2 c** and G2T1 was 2:1 (Figure 6 a, lane 8). The result implies that complex **2 c** might have a preference for the antiparallel conformation of quadruplex G2T1.

### Binding mode of complex 2 c toward G2T1

The results presented in the previous sections clearly indicate that complex **2 c** is a very good binder for antiparallel dimeric G‐quadruplex G2T1. We were therefore interested in further investigating its binding mode. In the UV/Vis titration experiments described above, the noticeable redshift observed at 360–390 nm suggests that complexes **2 c** and **1** interact with G2T1 through an end‐stacking mode.^35^ Both the UV/Vis titrations and CD melting studies clearly indicated that complex **2 c** has higher binding affinities toward G2T1 than complexes **2 a** and **2 b** with shorter polyether linkers (Table 1 and Figure 5 a). Moreover, the binding affinity of complex **2 c** towards the dimeric quadruplexes becomes progressively lower as the TTA‐linker becomes longer (Table 2). These results imply that the distance between the two nickel–salphen units in complex **2 c** matches the distance from the center of one G‐quartet plane to the center of another G‐quartet in G2T1, suggesting that this compound is likely to interact with the two G‐quadruplexes in G2T1.^24^

**Figure 7:**
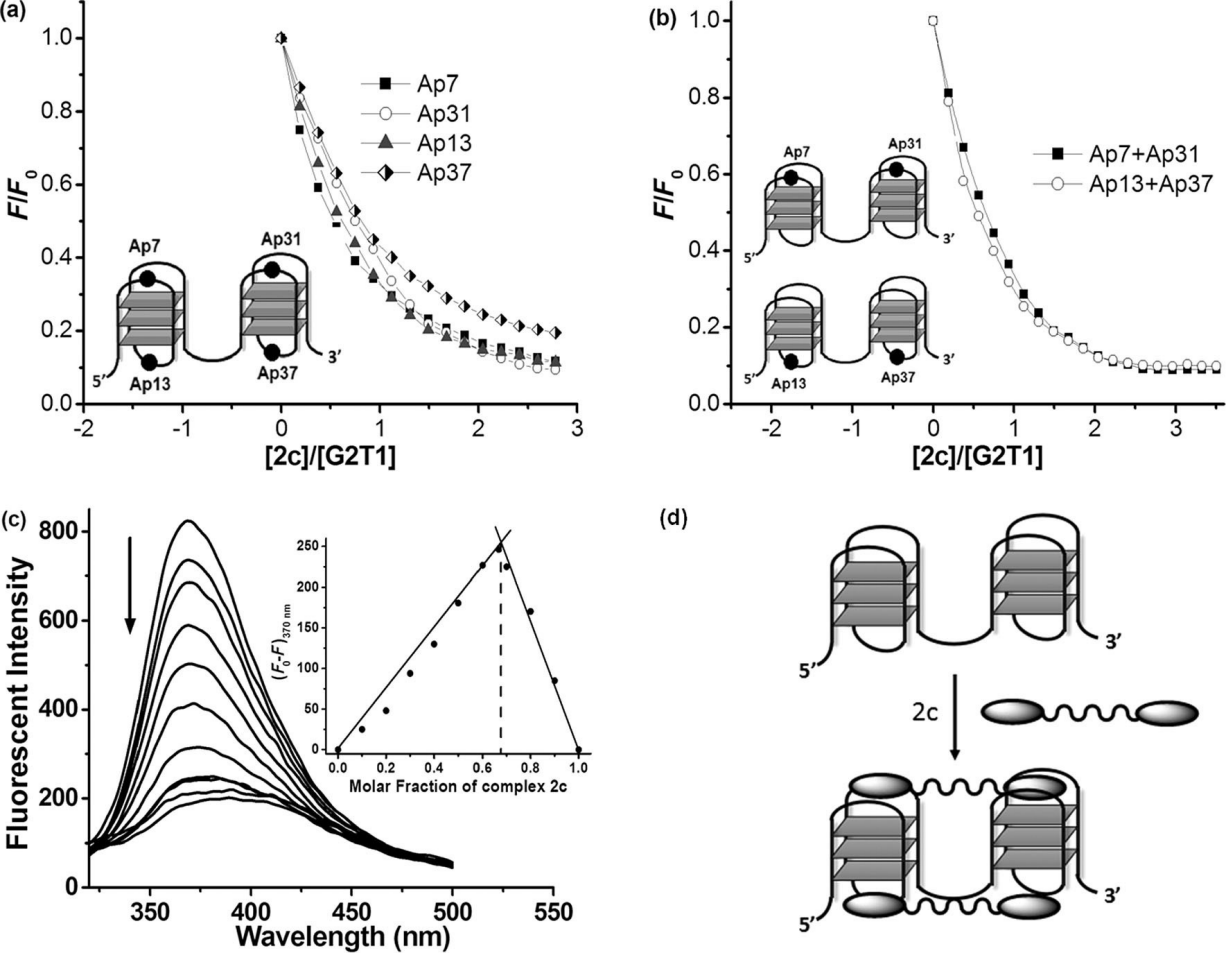
(a) Plot of normalized fluorescence intensity at 370 nm of 2‐Ap individually labeled G2T1 (Ap7, Ap13, Ap31, and Ap37, respectively) versus binding ratio of [**2 c**]/[G2T1]. (b) Plot of normalized fluorescence intensity at 370 nm of two 2‐Ap labeled G2T1 (Ap7+Ap31 and Ap13+Ap37) versus binding ratio of [**2 c**]/[G2T1]. Inset: illustration of the 2‐Ap position in G2T1. Experiments carried out in 10 mm Tris‐HCl, 100 mm NaCl (pH 7.04). (c) Fluorescence emission spectra of Ap31 titrated by complex **2 c**. Inset: Job's plot for complexation of **2 c** with Ap31. [**2 c**]+[Ap31]=3 μm, *λ*_ex_=305 nm. (d) Proposed binding interaction between nickel complex **2 c** and antiparallel dimeric quadruplex G2T1.

To study this possibility further, we carried out emission spectroscopic studies with G‐quadruplexes modified with 2‐aminopurine (Ap), a fluorescent adenine isomer that has been previously used to study the interaction of ligands with G‐quadruplexes.^24^, ^45^, ^46^, ^47^ In particular, we modified G2T1 with a single Ap base at positions 7, 13, 31, or 37 (named as Ap7, Ap13, Ap31, and Ap37, respectively; see Figure 7 a and Table 3). These positions were selected because they are located on the four different exposed G‐quartets in G2T1. Addition of complex **2 c** to the four different modified‐G2T1 sequences significantly decreased the fluorescent intensities of Ap7, Ap13, Ap31, and Ap37 (Figure 7 a), indicating that, upon binding with G2T1, complex **2 c** has considerable contact with the four Aps. This in turn suggests that the complex interacts with the four different tetrads in G2T1.

**Table 3:** DNA strands used in this work.

DNA	Sequence (from 5′ to 3′)	Structure
G1	AGGG(TTAGGG)_3_	G4 (monomeric)
G2T1	AGGG(TTAGGG)_7_	G4 (dimeric)
G2T2	AGGG(TTAGGG)_3_TTA(TTAGGG)_4_	G4 (dimeric)
G2T4	AGGG(TTAGGG)_3_(TTA)_3_(TTAGGG)_4_	G4 (dimeric)
G2T6	AGGG(TTAGGG)_3_(TTA)_5_(TTAGGG)_4_	G4 (dimeric)
Ap7	AGGGTT*Ap*GGG(TTAGGG)_6_^[a]^	G4 (dimeric)
Ap13	AGGGTTAGGGTT*Ap*GGG(TTAGGG)_5_	G4 (dimeric)
Ap31	AGGG(TTAGGG)_4_TT*Ap*GGG(TTAGGG)_2_	G4 (dimeric)
Ap37	AGGG(TTAGGG)5TT*Ap*GGGTTAGGG	G4 (dimeric)
Ap7+Ap31	AGGGTT*Ap*GGG(TTAGGG)_3_TT*Ap*GGG(TTAGGG)_2_	G4 (dimeric)
Ap13+Ap37	AGGGTTAGGGTT*Ap*GGG(TTAGGG)_3_TT*Ap*GGGTTAGGG	G4 (dimeric)

[a] *Ap*=2‐aminopurine.

To investigate further if **2 c** has a preference for G2T1′s external or internal tetrads, we modified the sequence with two Ap bases named Ap7+Ap31 and Ap13+Ap37 (Table 3). As can be seen in Figure 7 b, addition of **2 c** to either of the two doubly labeled G2T1 sequences, led to equally high quenching of Ap's emission. This observation would be consistent with two molecules of **2 c** interacting equally with each of the four G‐quartets of the two G‐quadruplex units in G2T1 as schematically shown in Figure 7 d. Therefore, we investigated the binding stoichiometry by titrating the Ap31‐labeled G2T1 with complex **2 c**, keeping the concentration sum of complex **2 c** and G2T1 constant, while varying the [**2 c**]/([**2 c**]+[G2T1]) ratios from 0 to 1.0. The Job's plot resulting from this titration (Figure 7 c) clearly shows a 2:1 binding between **2 c** and G2T1.^45^ This stoichiometry is consistent with our observations in the electrophoresis titration experiments (see Figure 6 a, lane 8).

Taken together, the UV/Vis titrations, CD‐melting, and fluorescence studies with Ap‐labelled G2T1, indicate that two molecules of complex **2 c** are likely to stack on the four end G‐quartets in G2T1. This binding mode—if confirmed by future structural studies—is different to most previously reported dimeric G‐quadruplex binders where the ligands interact at the cleft between the two G‐quadruplexes.^25^, ^26^, ^27^, ^29^

## Conclusion

In summary, three new dinickel–salphen complexes have been prepared and fully characterized. Using a combination of UV/Vis titrations, CD spectroscopy, CD‐melting assays, and electrophoresis, we have demonstrated that complex **2 c** (with the longest polyether linker) has high binding affinity towards dimeric quadruplex G2T1. This compound also displays the highest selectivity for G2T1 over G1, as compared to complexes **2 a** and **2 b** (with the shorter polyether linkers) and the monomeric nickel complex **1**. Fluorescent titration assays using Ap‐modified G2T1 suggest that two molecules of complex **2 c** may stack on the four end G‐quartets of the two well‐matched G‐quadruplex units in one G2T1. This work provides new insights into the binding properties of dimetallic complexes with dimeric quadruplex structures.

## Experimental Section

### General

^1^H NMR and ^13^C NMR spectra were recorded on either a Bruker Avance 400 MHz Ultrashield NMR spectrometer or a Bruker Avance 500 MHz NMR spectrometer. Mass spectrometric analysis was performed on a LCT Premier mass spectrophotometer. All chemicals were purchased from Sigma–Aldrich, BDH, or Apollo Scientific and used without further purification.

Oligonucleotides listed in Table 3 were purchased from Eurogentec (Belgium). Complexes **1** and **2 a**–**c** were dissolved in a mixture of DMSO (95 % by volume) and 1 mm HCl aqueous solution (5 % by volume) to give 2.0–3.0 mm stock solution. All solutions were diluted to 1 mm with DMSO before use. They were then further diluted using suitable buffer to the appropriate concentration.

### Synthesis

**1,5‐Bis(4‐amino‐3‐nitrophenyl‐5‐yl‐oxy) diethylene glycol ether (5 a)**: Compound **3 a** (400 mg, 1 mmol) was mixed with 4‐amino‐3‐nitrophenol **4** (364 mg, 2 mmol) and potassium carbonate (275 mg, 2 mmol) in dimethylformamide (5 mL), and the resulting reaction mixture was heated to 80 °C for 6 h. The mixture was then poured into water (50 mL) and filtered to obtain the crude product which was purified by chromatography on an aluminum oxide column, eluting with EtOH/EtOAc/petroleum ether (0.2/3/8, v/v/v), to afford compound **5 a** (175 mg, 46 %) as a red solid. ^1^H NMR (400 MHz, [D_6_]DMSO): *δ*=3.78 (t, *J=*4.2 Hz, 4 H, ‐CH_2_O‐), 4.07 (t, *J=*4.0 Hz, 4 H, ‐CH_2_O‐), 6.99 (d, *J=*9.2 Hz, 2 H, ArH), 7.18 (dd, *J=*9.2 Hz, *J=*2.4 Hz, 2 H, ArH), 7.26 (s, 4 H, ArH), 7.39 ppm (d, *J=*2.4 Hz, 2 H, ArH). ^13^C NMR (75.4 MHz, [D_6_]DMSO): *δ*=68.3, 69.2, 106.6, 121.2, 127.8, 129.5, 142.4, 148.8 ppm. ESI‐MS: *m*/*z* 379.1 ([*M*+H]^+^) and HRMS for C_16_H_18_N_4_O_7_ ([*M*+H]^+^) calcd: 379.1254; found: 379.1271.

**1,8‐Bis(4‐amino‐3‐nitrophenyl‐5‐yl‐oxy) triethylene glycol ether (5 b)**: This compound was prepared following the same procedure as the one described for compound **5 a**. The following amounts were used: compound **3 b** (458 mg, 1 mmol), 4‐amino‐3‐nitrophenol **4** (308 mg, 2 mmol), and potassium carbonate (276 mg, 2 mmol). Yield: 247 mg (59 %) as a red solid. ^1^H NMR (400 MHz, [D_6_]DMSO): *δ*=3.61 (s, 4 H, ‐CH_2_O‐), 3.73 (t, *J=*4.4 Hz, 4 H, ‐CH_2_O‐), 4.04 (t, *J=*4.8 Hz, 4 H, ‐CH_2_O‐), 6.99 (d, *J=*8.6 Hz, 2 H, ArH), 7.18 (dd, *J=*8.6 Hz, *J=*2.8 Hz, 2 H, ArH), 7.26 (s, 4 H, ArH), 7.39 ppm (d, *J=*2.4 Hz, 2 H, ArH). ^13^C NMR (75.4 MHz, [D_6_]DMSO): *δ*=68.2, 69.3, 70.4, 106.5, 121.2, 128.0, 129.5, 142.4, 148.8 ppm. ESI‐MS: *m*/*z* 422.1 ([*M*+H]^+^) and HRMS for C_18_H_22_N_4_O_8_ ([*M*+H]^+^) calcd: 423.1516; found: 423.1515.

**1,11‐Bis(4‐amino‐3‐nitrophenyl‐5‐yl‐oxy) tetraethylene glycol ether (5 c)**: This compound was prepared following the same procedure as the one described for compound **5 a**. The following amounts were used: compound **3 c** (825 mg, 1.64 mmol), 4‐amino‐3‐nitrophenol **4** (506 mg, 3.28 mmol) and potassium carbonate (453 mg, 3.28 mmol). Yield: 398 mg (52 %) as a red solid. ^1^H NMR (400 MHz, [D_6_]DMSO): *δ*=3.56 (s, 4 H, ‐CH_2_O‐), 3.57 (s, 4 H, ‐CH_2_O‐), 3.72 (t, *J*=4.4 Hz, 4 H, ‐CH_2_O‐), 4.04 (t, *J*=4.4 Hz, 4 H, ‐CH_2_O‐), 6.99 (d, *J*=8.6 Hz, 2 H, ArH), 7.19 (d, *J*=2.8 Hz, 2 H, ArH), 7.27 (s, 4 H, ArH), 7.38 ppm (d, *J*=2.8 Hz, 2 H, ArH). ^13^C NMR (75.4 MHz, [D_6_]DMSO): *δ*=68.2, 69.3, 70.4, 106.5, 121.2, 128.0, 129.5, 142.4, 148.8 ppm. ESI‐MS: *m*/*z* 489.2 ([*M*+Na]^+^) and HRMS for C_20_H_26_N_4_O_9_ ([*M*+Na]^+^) calcd: 489.1597; found: 489.1598.

**1,5‐Bis(3,4‐diaminophenyl‐5‐yl‐oxy) diethylene glycol ether (6 a)**: Hydrogen was bubbled through a stirred mixture of compound **5 a** (50 mg, 0.132 mmol), Pd−C (25 mg, 10 %) and methanol (20 mL) under reflux for 2 h. After filtration, the filtrate was evaporated under reduced pressure and the resulting oil dried by flushing N_2_ through to afford compound **5 a** (32.6 mg, 77 %) as a brown oily liquid.^1^H NMR (400 MHz, [D_6_]DMSO): *δ*=3.71 (t, *J=*4.6 Hz, 4 H, ‐CH_2_O‐), 3.90 (t, *J=*4.6 Hz, 4 H, ‐CH_2_O‐), 4.00 (s, 4 H, ‐NH_2_), 4.48 (s, 4 H, ‐NH_2_), 5.99 (dd, *J=*8.0 Hz, *J*=2.4 Hz, 2 H, ArH), 6.17 (d, *J=*2.8 Hz, 2 H, ArH), 6.41 ppm (d, *J=*8.0 Hz, 2 H, ArH). ^13^C NMR (75.4 MHz, [D_6_]DMSO): *δ*=67.8, 69.8, 102.4, 102.9, 115.7, 129.1, 137.0, 151.7 ppm. ESI‐MS: *m*/*z* 319.2 ([*M*+H]^+^) and HRMS for C_16_H_22_N_4_O_3_ ([*M*+H]^+^) calcd: 319.1770; found: 319.1783.

**1,8‐Bis(3, 4‐diaminophenyl‐5‐yl‐oxy)‐3,6‐dioxyoctane (6 b)**: This compound was prepared following the same procedure as the one described for compound **6 a**. The following amounts were used: compound **5 b** (50 mg, 0.138 mmol) and Pd−C (25 mg, 10 %). Yield: 34.8 mg (81 %) as a brown oil liquid. ^1^H NMR (400 MHz, [D_6_]DMSO): *δ*=3.59 (s, 4 H, ‐CH_2_O‐), 3.67 (t, *J=*4.8 Hz, 4 H, ‐CH_2_O‐), 3.88 (t, *J=*4.4 Hz, 4 H, ‐CH_2_O‐), 3.99 (s, 4 H, ‐NH_2_), 4.48 (s, 4 H, ‐NH_2_), 5.98 (dd, *J=*8.2 Hz, *J=*2.8 Hz, 2 H, ArH), 6.17 (d, *J=*2.4 Hz, 2 H, ArH), 6.40 ppm (d, *J=*8.2 Hz, 2 H, ArH). ^13^C NMR (75.4 MHz, [D_6_]DMSO): *δ*=67.6, 69.7, 70.4, 102.4, 102.8, 115.7, 129.1, 137.2, 151.7 ppm. HRMS (ESI^+^) for C_18_H_26_N_4_O_4_ ([*M*+H]^+^) calcd: 363.2032; found: 363.2044.

**1,11‐Bis(3,4‐diaminophenyl‐5‐yl‐oxy)‐3,6,9‐trioxaundecane (6 c)**: This compound was prepared following the same procedure as the one described for compound **6 a**. The following amounts were used: compound **5 c** (52.6 mg, 0.113 mmol) and Pd−C (25 mg, 10 %). Yield: 42 mg (92 %) as a brown oily liquid. ^1^H NMR (400 MHz, [D_6_]DMSO): *δ*=3.55 (q, 4 H, ‐CH_2_O‐), 3.56 (q, 4 H, ‐CH_2_O‐), 3.66 (t, *J=*4.8 Hz, 4 H, ‐CH_2_O‐), 3.87 (t, *J=*4.8 Hz, 4 H, ‐CH_2_O‐), 3.99 (s, 4 H, ‐NH_2_), 4.48 (s, 4 H, ‐NH_2_), 5.98 (dd, *J=*8.4 Hz, *J=*2.4 Hz, 2 H, ArH), 6.16 (d, *J=*2.8 Hz, 2 H, ArH), 6.40 ppm (d, *J=*8.4 Hz, 2 H, ArH). ^13^C NMR (75.4 MHz, [D_6_]DMSO): *δ*=67.6, 69.7, 70.4, 102.4, 102.9, 115.7, 137.0, 158.5 ppm. HRMS (ESI^+^) for C_20_H_30_N_4_O_5_ ([*M*+H]^+^) calcd: 407.2294; found: 407.2294.

**1,5‐Bis [*N***,***N*** **′‐bis[4‐[[1‐(2‐ethyl)piperidine]oxy]salicylidene]‐4‐oxy‐1,2‐phenylene‐diamine]‐3‐oxypentane‐bisnickel(II) (2 a)**: Compound **6 a** (32.6 mg, 0.1024 mmol) and compound **7** (103 mg, 0.4096 mmol) were dissolved in ethanol and heated at reflux for 2 h. Ni(OAc)_2_⋅4 H_2_O (50.97 mg, 0.2048 mmol) was then added to this solution and the reaction mixture was refluxed for another 16 h. The solvent was removed under reduced pressure yielding a red solid. The solid was then recrystallized from CH_2_Cl_2_‐pentane to yield **2 a** as a red‐black solid. Yield: 65 mg, 46 %. ^1^H NMR (400 MHz, [D_6_]DMSO): *δ*=1.39 (br, 8 H, piperidine‐H), 1.51 (br, 16 H, piperidine‐H), 2.43 (br, 16 H, piperidine‐H), 2.64 (br, 8 H, ‐CH_2_N‐), 3.84 (br, 4 H, ‐CH_2_O‐), 4.05 (br, 8 H, ‐CH_2_O‐), 4.20 (br, 4 H, ‐CH_2_O‐), 6.24–6.30 (br, 8 H, ArH), 6.82 (br, 2 H, ArH), 7.34 (br, 4 H, ArH), 7.49 (br, 2 H, ArH), 7.89 (br, 2 H, ArH), 8.35 (s, 2 H, ‐CH=*N*‐), 8.40 ppm (s, 2 H, ‐CH=*N*‐). ^13^C NMR (75.4 MHz, 353 K, [D_6_]DMSO): *δ*=23.5, 25.2, 54.0, 54.3, 56.7, 65.6, 67.9, 68.9, 101.0, 101.6, 106.4, 107.0, 113.5, 114.6, 114.7, 115.1, 134.3, 134.6, 135.9, 143.0, 152.4, 153.7, 157.4, 163.8, 164.3, 166.4, 167.3 ppm. MALDI‐TOF: *m*/*z* 1379.9 ([*M*+Na]^+^) for C_72_H_86_N_8_Ni_2_O_11_. Elemental analysis calcd (%) for C_72_H_86_N_8_Ni_2_O_11_⋅2 CH_2_Cl_2_ (mol. mass: 1536.8 g mol^−1^): C 58.21, H 5.94, N 7.34; found: C 58.64, H 6.45, N 7.45.

**1,8‐Bis [*N***,***N*** **′‐bis[4‐[[1‐(2‐ethyl)piperidine]oxy]salicylidene]‐4‐oxy‐1,2‐phenylene ‐diamine]‐3,6‐dioxyoctane‐bisnickel(II) (2 b)**: This compound was prepared following the same procedure as the one described for compound **2 a**. The following amounts were used: compounds **6 b** (34.4 mg, 0.095 mmol), **7** (94.6 mg, 0.38 mmol), and Ni(OAc)_2_⋅4 H_2_O (47.2 mg, 0.19 mmol). Yield: 120 mg (90 %) as a red‐black solid. ^1^H NMR (400 MHz, [D_6_]DMSO): *δ*=1.38 (br, 8 H, piperidine‐H), 1.48 (br, 16 H, piperidine‐H), 2.39 (br, 16 H, piperidine‐H), 2.61 (br, 8 H, ‐CH_2_N‐), 3.63 (br, 4 H, ‐CH_2_O‐), 3.74 (br, 4 H, ‐CH_2_O‐), 3.92 (br, 4 H, ‐CH_2_O‐), 4.04 (br, 4 H, ‐CH_2_O‐), 4.09 (br, 4 H, ‐CH_2_O‐), 6.17–6.30 (br, 8 H, ArH), 6.78 (br, 2 H, ArH), 7.24 (d, *J=*6.4 Hz, 2 H, ArH), 7.30 (d, *J=*8.4 Hz, 2 H, ArH), 7.43 (br, 2 H, ArH), 7.79 (d, *J=*7.2 Hz, 2 H, ArH), 8.19 (s, 2 H, ‐CH=*N*‐), 8.31 ppm (s, 2 H, ‐CH=*N*‐). ^13^C NMR (75.4 MHz, 353 K, [D_6_]DMSO): *δ*=23.4, 25.1, 53.8, 56.5, 56.6, 65.5, 67.6, 68.6, 69.7, 100.7, 101.5, 106.3, 106.8, 113.1, 114.5, 114.6, 115.0, 134.2, 134.5, 135.7, 143.0, 152.2, 153.7, 157.4, 163.7, 164.1, 166.2, 167.2. MALDI‐TOF: *m*/*z* 1401.6 ([*M*+H]^+^) for C_74_H_90_N_8_Ni_2_O_12_. Elemental analysis calcd (%) for C_74_H_90_N_8_Ni_2_O_12_⋅3 CH_2_Cl_2_ (mol. mass: 1655.7 g mol^−1^): C 55.86, H 5.84, N 6.77; found: C 55.82, H 5.99, N 6.91.

**1,11‐Bis [*N***,***N*** **′‐bis[4‐[[1‐(2‐ethyl)piperidine]oxy]salicylidene]‐4‐oxy‐1,2‐phenylene‐diamine]‐3,6,9‐trioxaundecane‐bisnickel(II) (2 c)**: This compound was prepared following the same procedure as the one described for compound **2 a**. The following amounts were used: compounds **6 c** (42 mg, 0.1033 mmol), **7** (103 mg, 0.41 mmol), and Ni(OAc)_2_⋅4 H_2_O (51.41 mg, 0.21 mmol). Yield: 124 mg (83 %) as a red‐black solid. ^1^H NMR (400 MHz, [D_6_]DMSO): *δ*=1.38 (br, 8 H, piperidine‐H), 1.49 (br, 16 H, piperidine‐H), 1.91(s, 9 H, CH_3_COO‐), 2.41 (br, 16 H, piperidine‐H), 2.63 (br, 8 H, ‐CH_2_N‐), 3.57 (br, 4 H, ‐CH_2_O‐), 3.59 (br, 4 H, ‐CH_2_O‐), 3.75 (br, 4 H, ‐CH_2_O‐), 3.96 (br, 4 H, ‐CH_2_O‐), 4.04 (br, 4 H, ‐CH_2_O‐), 4.09 (br, 4 H, ‐CH_2_O‐), 6.19–6.30 (br, 8 H, ArH), 6.78 (d, *J*=8.4 Hz, 2 H, ArH), 7.28 (d, *J*=8.8 Hz, 2 H, ArH), 7.33 (d, *J*=8.8 Hz, 2 H, ArH), 7.50 (br, 2 H, ArH), 7.81 (d, *J*=9.2 Hz, 2 H, ArH), 8.24 (s, 2 H, ‐CH=*N*‐), 8.42 (s, 2 H, ‐CH=*N*‐). ^13^C NMR (75.4 MHz, [D_6_]DMSO): *δ*=23.4, 25.1, 53.8, 56.6, 65.5, 67.7, 68.7, 69.5, 100.7, 101.4, 106.3, 106.9, 113.1, 114.6, 115.0, 134.2, 134.6, 135.7, 143.1, 152.2, 153.9, 157.5, 163.6, 164.2, 166.2, 167.2. MALDI‐TOF: *m*/*z* 1467.9 ([*M*+Na]^+^) for C_76_H_94_N_8_Ni_2_O_13_. Elemental analysis calcd (%) for C_76_H_94_N_8_Ni_2_O_13_⋅2 CH_2_Cl_2_⋅3 CH_3_COOH (mol. mass: 1795.01 g mol^−1^): C 56.1, H 6.18, N, 6.24; found: C 55.75, H 6.09, N 6.30.

### UV/Vis titration assays

The corresponding oligonucleotides, human telomeric G1 and G2T1, were dissolved in 10 mm Tris‐HCl, pH 7.04, and 100 mm KCl (or NaCl) buffer to yield a 500 μm solution. The oligonucleotide was annealed by heating to 95 °C for 10 min and then cooled to room temperature overnight. The UV/Vis spectra were recorded on a PerkinElmer Lambda 25 spectrometer. To determine the binding constants of the selected complexes with DNA including G2T1, G1, and CT DNA, the complex (20 μm) was titrated with concentrated solutions of DNA (500 μm) in 100 mm KCl (or NaCl) buffer. A 1 cm path‐length quartz cuvette was used to carry out the measurements. The apparent binding constants (*K*_a_ values) were obtained by fitting the data to a reciprocal plot of *D*/Δ*ϵ*_ap_ versus *D* using the following equation: *D*/Δ*ϵ*_ap_=*D*/Δ*ϵ*+1/(Δ*ϵ×K*_a_).^35^, ^48^ The concentration of DNA (*D*) is expressed in terms of base pairs (determined by measuring the absorption at 260 nm and the appropriate extinction coefficients); the apparent molar extinction coefficient *ϵ*_a_=*A*_observed_/[Complex], Δ*ϵ*_ap_=[*ϵ*_a_−*ϵ*_f_], and Δ*ϵ*=[*ϵ*_b_−*ϵ*_f_]; *ϵ*_b_ is the extinction coefficient of the DNA bound complex, and *ϵ*_f_ is the extinction coefficient of the free complex.

### CD spectroscopy

The oligonucleotides G1 and G2T1 were dissolved in Milli Q. water to yield a 1 mm stock solution. They were then diluted using 10 mm Tris‐HCl and 100 mm NaCl or KCl (pH 7.04) buffer to 10 μm. Prior to use in the CD assay, the DNA solution was either annealed or remained nonannealed. The DNA solution was annealed by heating the solution to 95 °C for 10 min and then cooling to room temperature overnight. The CD spectra were measured in a strain‐free 10 mm×2 mm rectangular cell path length cuvette. The CD spectra were measured in the spectral range of 600–200 nm. The following CD spectra were recorded: (1) CD spectra of annealed G2T1 (2.5 μm) in 10 mm Tris‐HCl and 100 mm NaCl (pH 7.04) with complexes **1** and **2 a**–**c**; (2) CD spectra of annealed G2T1 (2.5 μm) in 10 mm Tris‐HCl and 100 mm KCl (pH 7.04) with complexes **1** and **2 a**–**c**; (3) CD spectra of nonannealed G2T1 (2.5 μm) in 10 mm Tris‐HCl (pH 7.04) with complexes **1** and **2 a**–**c**.

### CD‐melting

The oligonucleotides, G1, G2T1, G2T2, G2T4, and G2T6, were dissolved in Milli Q. water to yield a 1 mm stock solution. They were then diluted using 10 mm Tris‐HCl and 100 mm KCl or NaCl (pH 7.04) to 10 μm. Prior to use in the CD assay, the DNA solution was annealed by heating the solution to 95 °C for 10 min and then cooling to room temperature overnight. The preparation of the solutions was similar to the procedure described for the UV/Vis titrations. CD spectra were measured in the wavelength range of 230–340 nm using a quartz cuvette with 1.0 nm path length. The scanning speed was 100 nm min^−1^, and the response time was 2 s. CD‐melting was monitored at 295 nm at a heating rate of 1 °C min^−1^ from 25 to 95 °C. The melting temperature (*T*_m_) was determined from the melting profiles with the software origin 8.0.

### Gel electrophoresis

The oligonucleotides G2T1 and G1 were dissolved in Milli Q. water to yield a 1 mm stock solution. They were then diluted to 20 μm with 10 mm Tris‐HCl and 100 mm NaCl (pH 7.04). The DNA solutions were annealed at 95 °C for 10 min, gradually cooled to room temperature, and incubated at 4 °C overnight. The final loading sample was prepared by mixing complex **2 c** or **1** (100 μm) with the annealed DNA samples, followed by incubation at 4 °C for 3 h. Native gel electrophoresis was carried out on acrylamide gel (15 %), run at 0 °C in 1×TBE buffer (pH 8.3) and was stained by ethidium bromide. DNA binding selectivity was analyzed with Alpha Hp 3400 fluorescent and visible light digitized image analyzer.

### Fluorescence spectroscopy

The Ap‐labeled oligonucleotides were dissolved in 10 mm Tris‐HCl and 100 mm NaCl (pH 7.04) buffer to yield a 5 μm solution. The oligonucleotide was annealed by heating to 95 °C for 10 min and then cooled to room temperature overnight. Fluorescent measurements were carried out on a PerkinElmer spectrofluorometer at 25 °C.^24^, ^45^ The fluorescence spectra were measured at *λ*_ex_/*λ*_em_=305/370 nm with ex/em=10/10 nm. The DNA solution (5 μm) was titrated with a concentrated solution of 1 mm complex **2 c** or **1** (buffer: 10 mm Tris‐HCl, 100 mm NaCl, pH 7.04). For the binding stoichiometry assays between complex **2 c** and Ap31‐G2T1, the spectra were recorded by keeping the concentration sum of complex **2 c** and Ap31 constant ([**2 c**]+[Ap31]=3 μm), while increasing the [**2 c**]/([**2 c**]+[Ap31]) ratio. The stoichiometric ratio between complex **2 c** and G2T1 was obtained by plotting (*F*_0_−*F*) at 370 nm against the [**2 c**]/([**2 c**]+[Ap31]) ratios varying from 0 to 1.0. *F*_0_ is the fluorescent intensity of the Ap31 solution; *F* is the fluorescent intensity of the mixture of complex **2 c** and Ap31.

## Conflict of interest

The authors declare no conflict of interest.

## Supporting information

As a service to our authors and readers, this journal provides supporting information supplied by the authors. Such materials are peer reviewed and may be re‐organized for online delivery, but are not copy‐edited or typeset. Technical support issues arising from supporting information (other than missing files) should be addressed to the authors.

Supplementary
