# Supplementary material for: Dinickel–Salphen Complexes as Binders of Human Telomeric Dimeric G‐Quadruplexes
Source: Chemistry. 2017 Mar 24;23(19):4713–22. doi: 10.1002/chem.201700276 (PMC5516229; doi:10.1002/chem.201700276)
Supplement: Supplementary file 1 — Supplementary [file CHEM-23-4713-s001.pdf]

CHEMISTRY
A European Journal

Supporting Information

Dinickel–Salphen Complexes as Binders of Human Telomeric Dimeric G-Quadruplexes

Chun-Qiong Zhou,[a, b] Ting-Cong Liao,[b] Zi-Qi Li,[b] Jorge Gonzalez-Garcia,[a] Matthew Reynolds,[a] Min Zou,[b] and Ramon Vilar*[a]

chem_201700276_sm_miscellaneous_information.pdf

Table S1. Apparent affinity constants (*K*a's, M^{-1}) of complexes **2a-c** and **1** for quadruplexes G2T1, G1 and CT DNA in 10 mM Tris-HCl, 100 mM KCl (pH 7.04) by UV-Vis spectroscopy.

Complex	K_a (G2T1)	K_a (G1)	K_a (CT DNA)	Selectivity for G2T1 vs. G1	Selectivity for G2T1 vs. CT-DNA
**2a**	[a] $1.19\pm0.16\times10^6$	[a] $2.39\pm0.35\times10^6$	$2.54\pm0.24\times10^5$	1	5
**2b**	[a] $2.52\pm0.24\times10^6$	[a] $3.53\pm0.33\times10^6$	$2.05\pm0.08\times10^5$	1	12
**2c**	[b] $1.37\pm0.25\times10^7$	[b] $2.19\pm0.32\times10^6$	$1.06\pm0.11\times10^5$	6	129
**1**	[a] $1.50\pm0.22\times10^7$	[b] $1.15\pm0.18\times10^7$	$1.20\pm0.15\times10^5$	1	125

[a] Absorption measured at 310 nm; [b] Absorption measured at 370 nm.

Table S2. Binding constants (*K*'s, M^{-1}) and ΔT_m (°C) of previously reported binders towards dimeric quadruplex G2T1 DNA.

Binder	*K*	ΔT_m	References
Cyclic helicene M1	2.31×10^6	/	[1]
QATPE [a]	8.94×10^6	6.6	[2]
TMPipEOPP (p- and m-) [b]	$(1.05\sim2.53)\times10^6$	5.1~13	[3]
RHPS4 [c] and DR4-47 [d]	/	20.5~28.5	[4]
EPI [e]	2.60×10^7	/	[5]
Ni-M [f]	4.6×10^7	14	[6]
Dimeric berberine	$(2.0\sim2.4)\times10^7$	-0.5	[7]

[a]QATPE = 1, 1, 2, 2-Tetrakis{4-[(trimethylammonium)butoxy]phenyl}tetraphenylethene tetrabromide; [b] TMPipEOPP = cationic porphyrin derivative; [c] RHPS4 = fluoroquinolinoacri-dinium cation; [d] DR4-47 = Hybrid oxazole-triazole ligand; [e] EPI = epiberberine; [f] Ni-M = Zinc-finger like nanosized chiral Ni(II)-supramolecular complex.

[1] K. Shinohara, Y. Sannohe, S. Kaieda, K. Tanaka, H. Osuga, H. Tahara, Y. Xu, T. Kawase, T. Bando, H. Sugiyama, *J. Am. Chem. Soc.* **2010**,*132*, 3778-3782.

[2] Q. Zhang, Y. -C. Liu, D. -M. Kong, D. -S. Guo, *Chem. Eur. J.* **2015**, *21*, 13253-13260.

[3] a) X. -X. Huang, L. -N. Zhu, B. Wu, Y. -F. Huo, N. -N. Duan, D. -M. Kong, *Nucleic Acids Res.* **2014**, *42*, 8719-8731; b) L. -N. Zhu, B. Wu, D. -M. Kong, *Nucleic Acids Res.* **2013**, *41*, 4324-4335.

[4] A. R. O. Cousins, D. Ritson, P. Sharma, M. F. G. Stevens, J. E. Moses, M. S. Searle, *Chem. Commun.* **2014**, *50*, 15202-15205.

[5] L. Zhang, H. Liu, Y. Shao, C. Lin, H. Jia, G. Chen, D. Yang, Y. Wang, *Anal. Chem.* **2015**, *87*, 730-737.

[6] C. Q. Zhao, L. Wu, J. S. Ren, Y. Xu, X. G. Qu, *J. Am. Chem. Soc.* **2013**, *135*, 18786-18789.

[7] C. -Q. Zhou, J. -W. Yang, C. Dong, Y. -M. Wang, B. Sun, J. -X. Chen, Y. -S. Xu, W. -H. Chen, *Org. Biomol. Chem.* **2016**, *14*, 191-197.

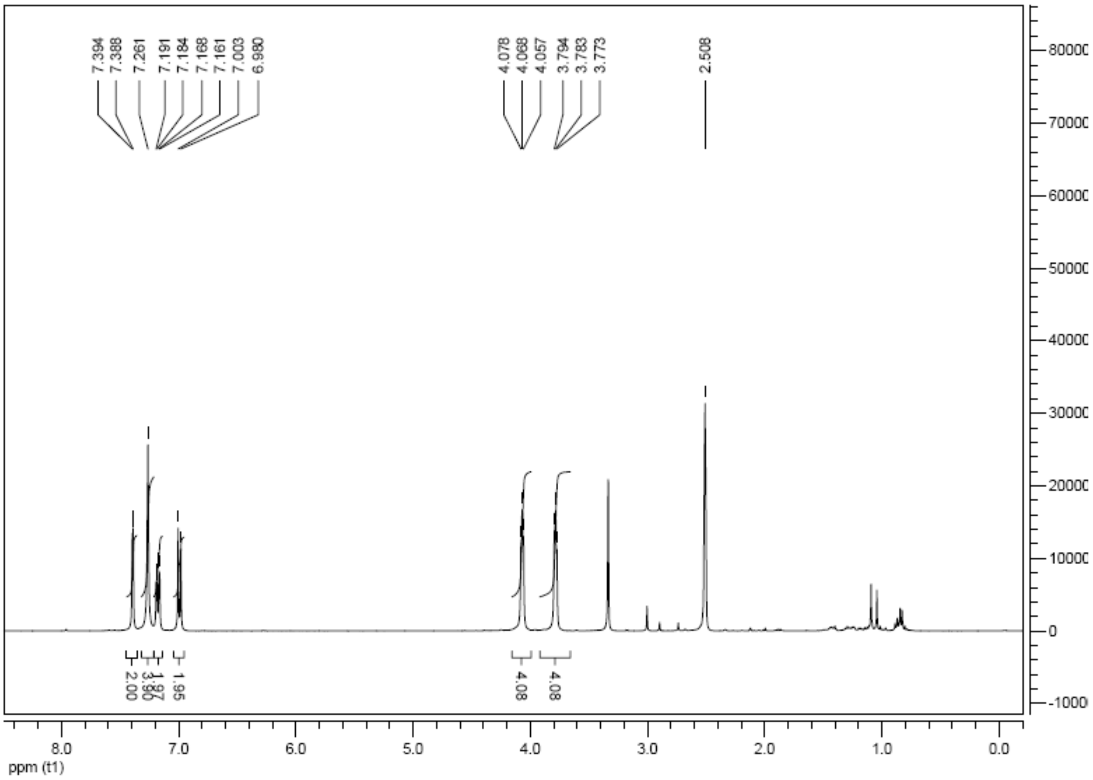

Figure S1. ^{1}H NMR (400 MHz) of compound **5a** in d_6-DMSO.

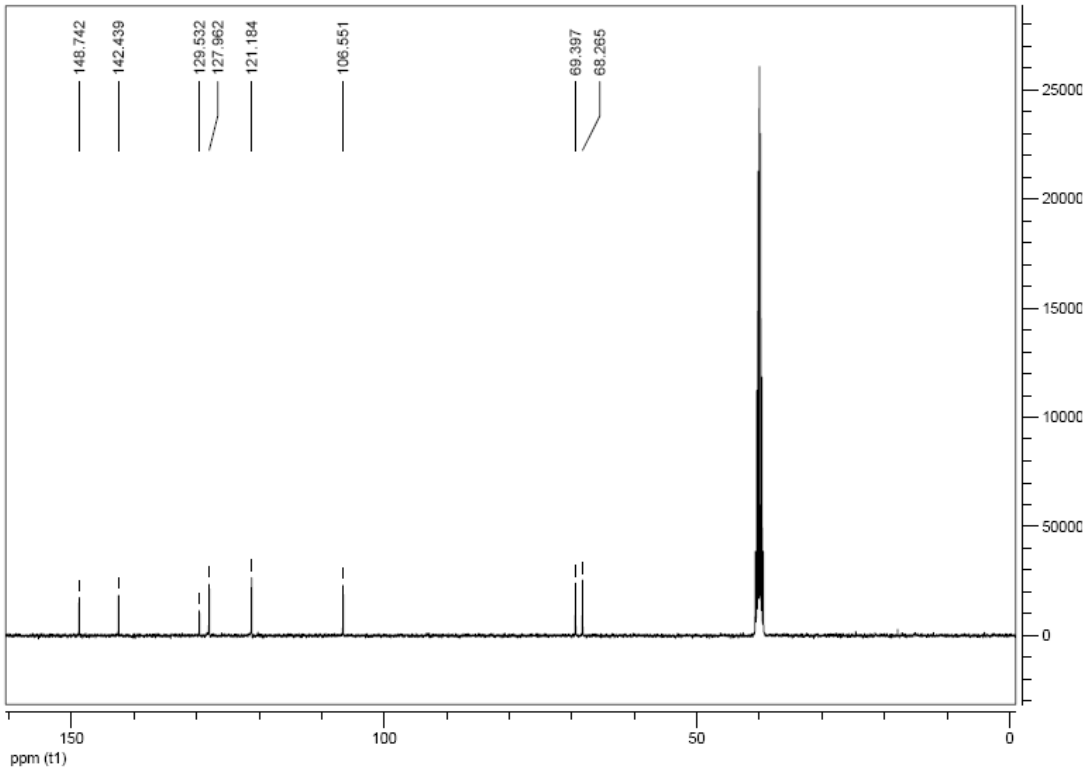

Figure S2. ^{13}C NMR (75.4 MHz) of compound **5a** in d_6-DMSO.

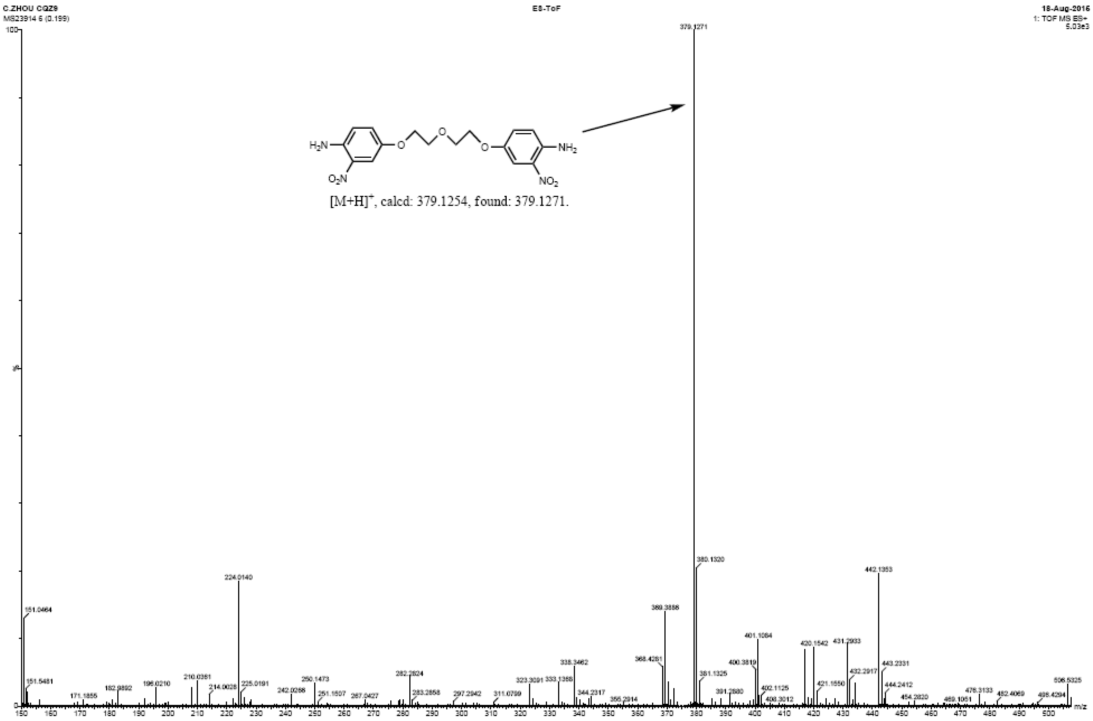

Figure S3. HR-ESI-MS of compound **5a**.

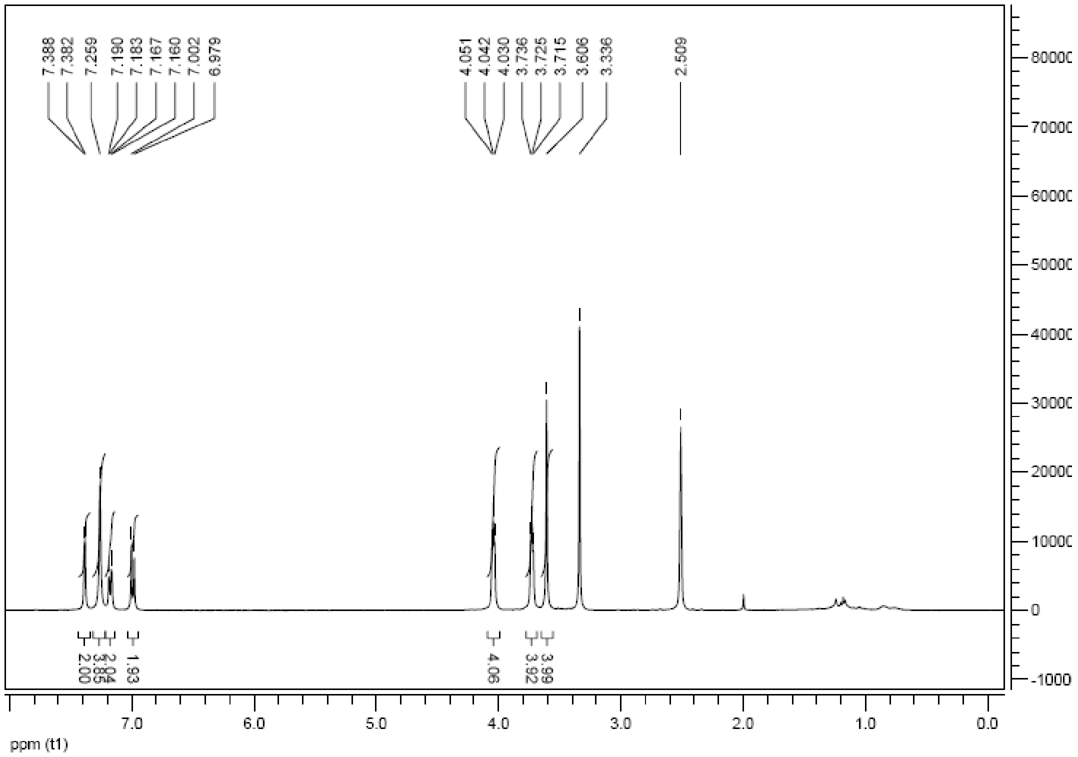

Figure S4. ^{1}H NMR (400 MHz) of compound **5b** in d_6-DMSO.

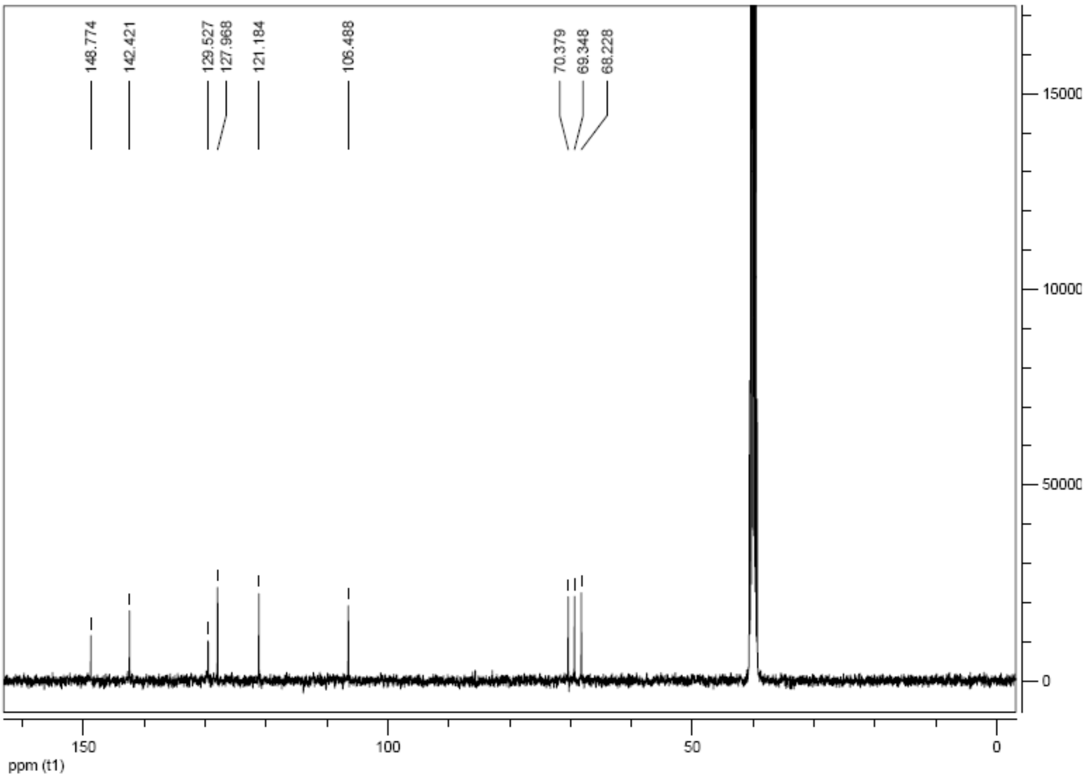

Figure S5. ^{13}C NMR (75.4 MHz) of compound **5b** in d_6-DMSO.

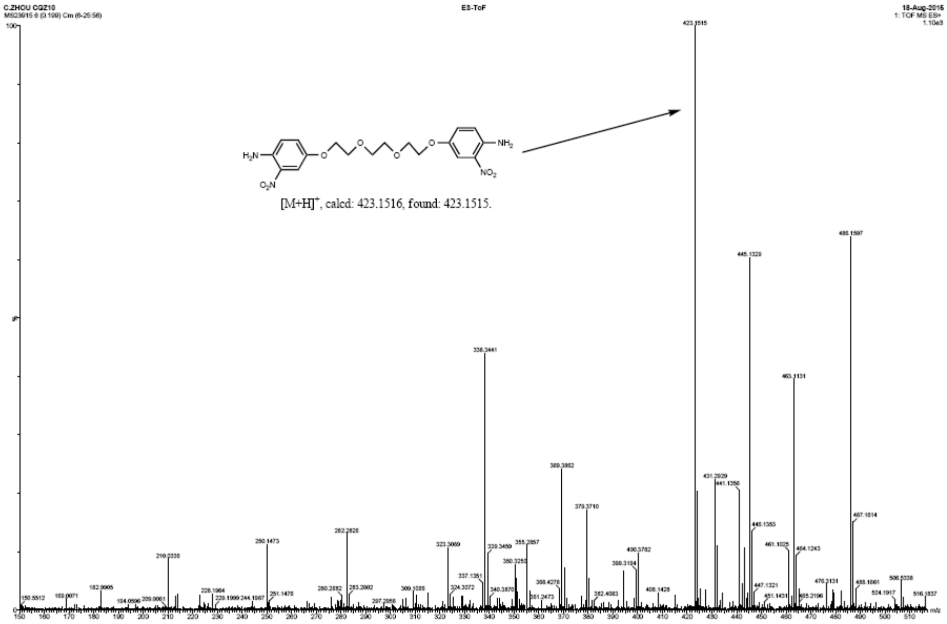

Figure S6. HR-ESI-MS of compound **5b**.

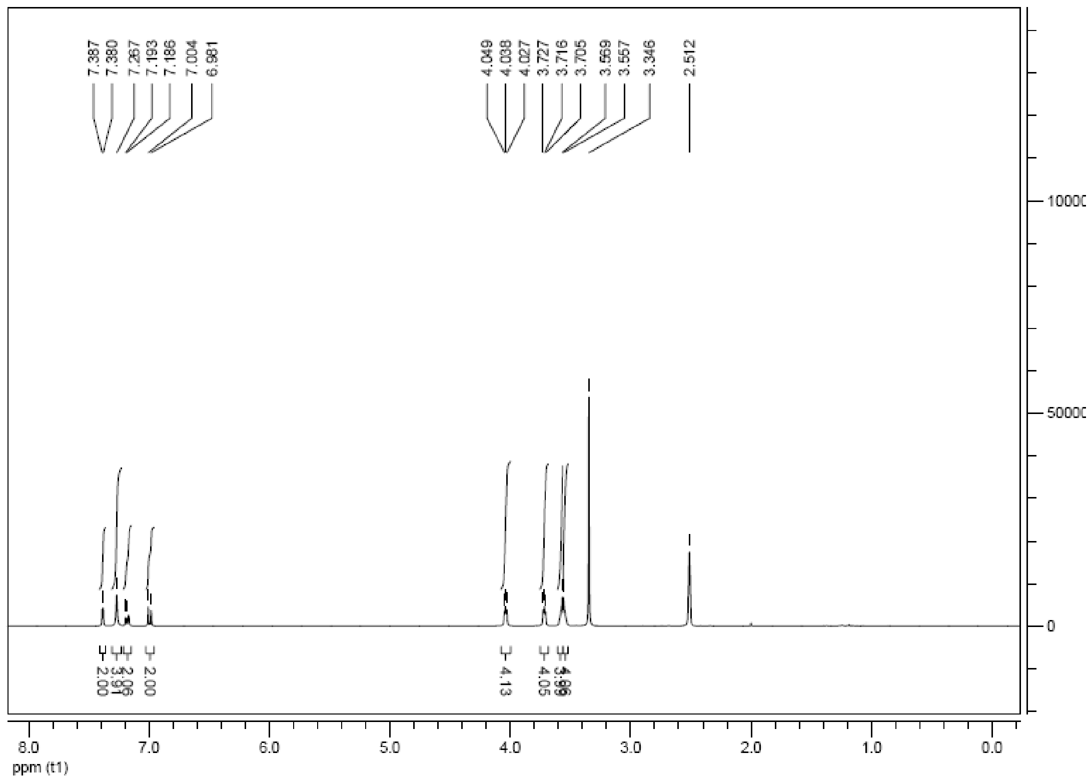

Figure S7. ^{1}H NMR (400 MHz) of compound **5c** in d_6-DMSO.

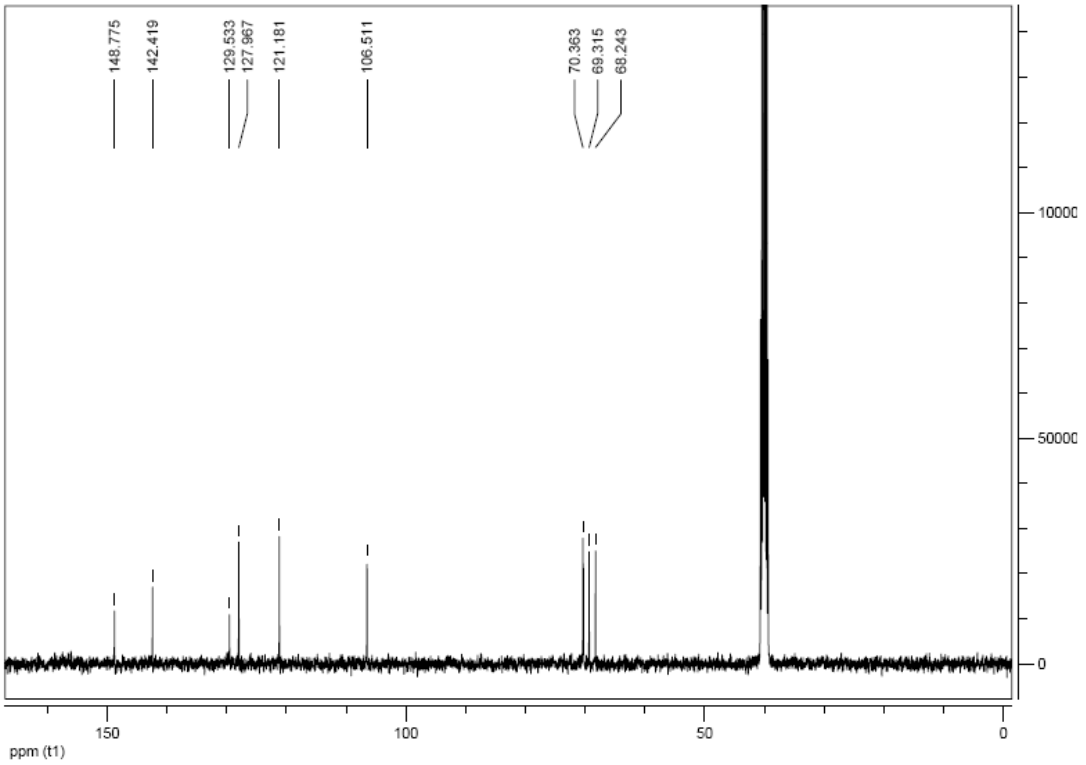

Figure S8. ^{13}C NMR (75.4 MHz) of compound **5c** in d_6-DMSO.

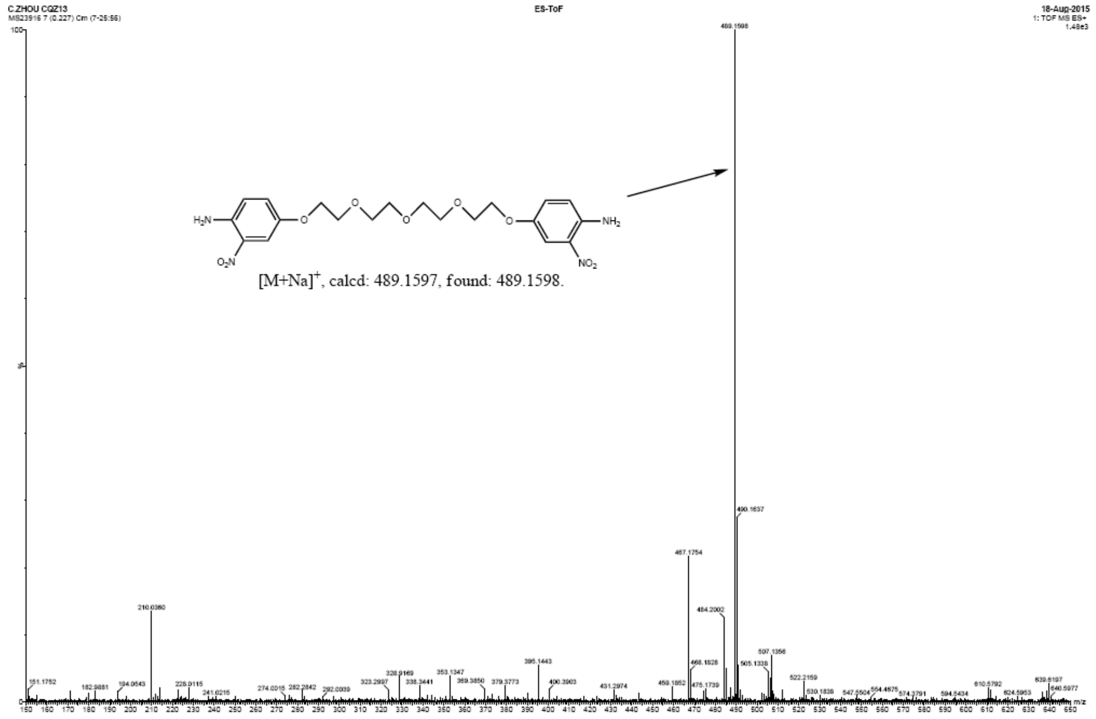

Figure S9. HR-ESI-MS of compound **5c**.

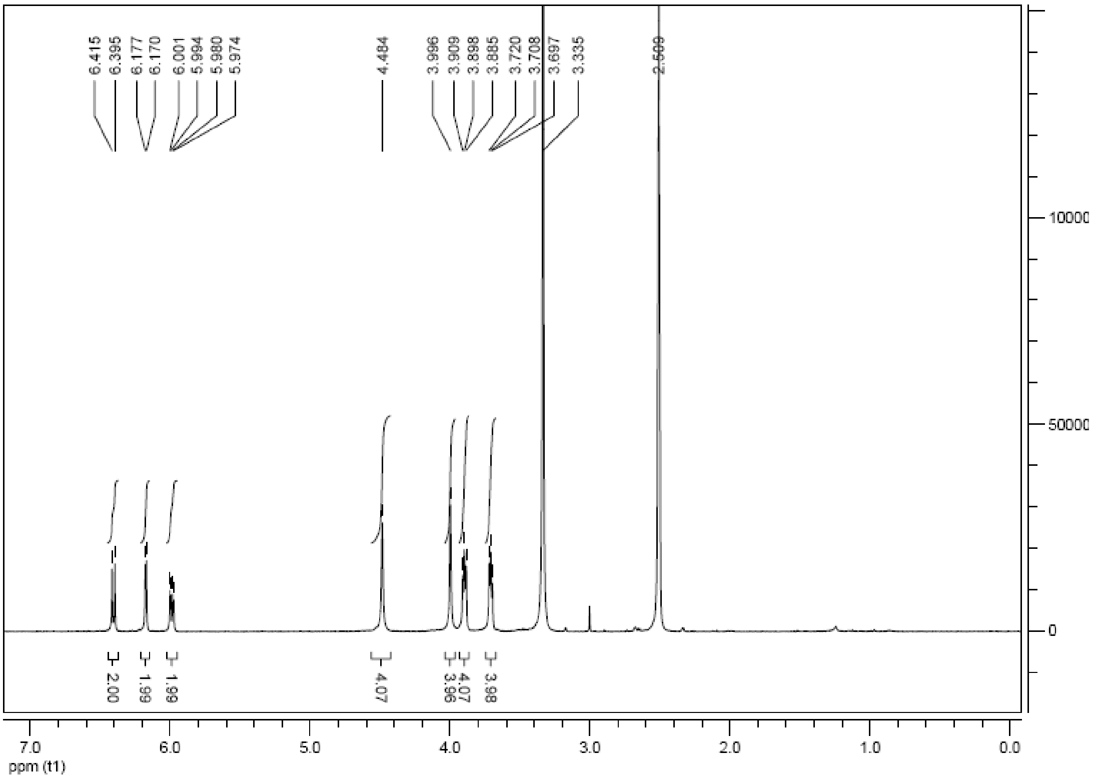

Figure S10. ^{1}H NMR (400 MHz) of compound **6a** in d_6-DMSO.

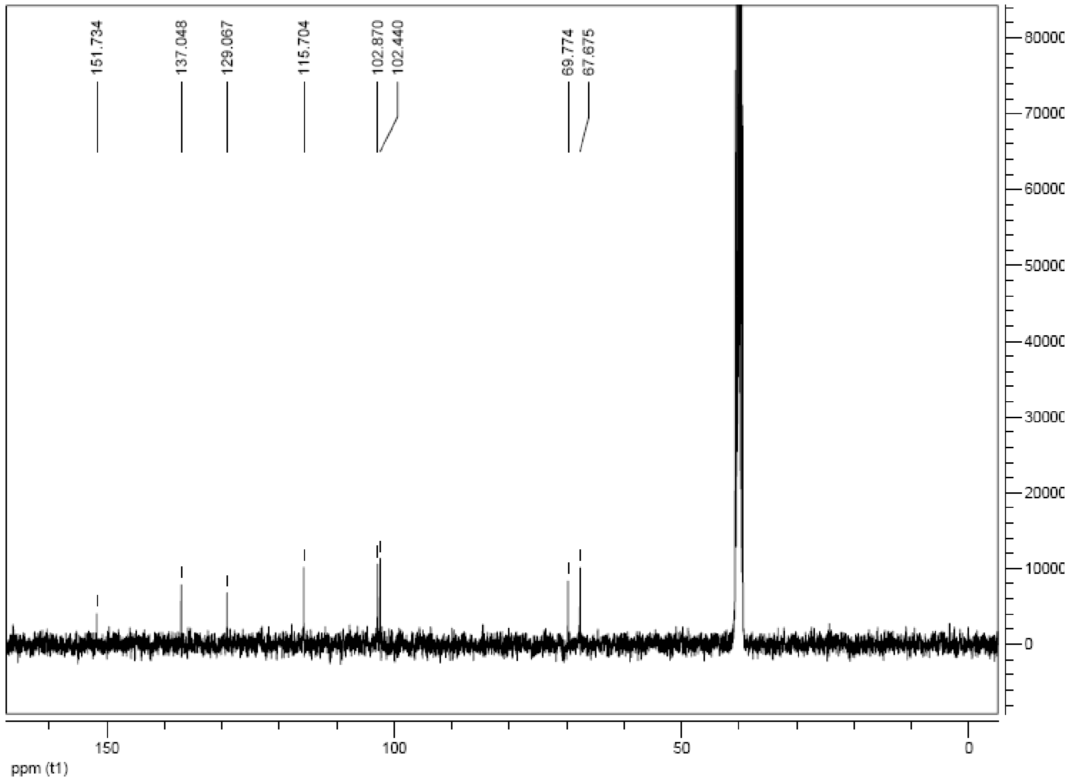

Figure S11. ^{13}C NMR (75.4 MHz) of compound **6a** in d_6-DMSO.

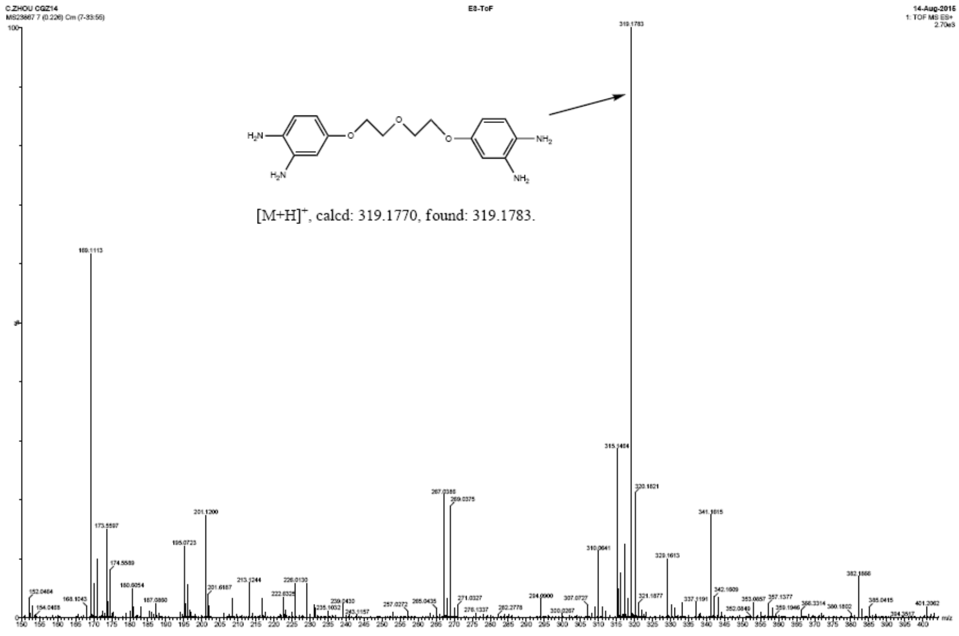

Figure S12. HR-ESI-MS of compound **6a**.

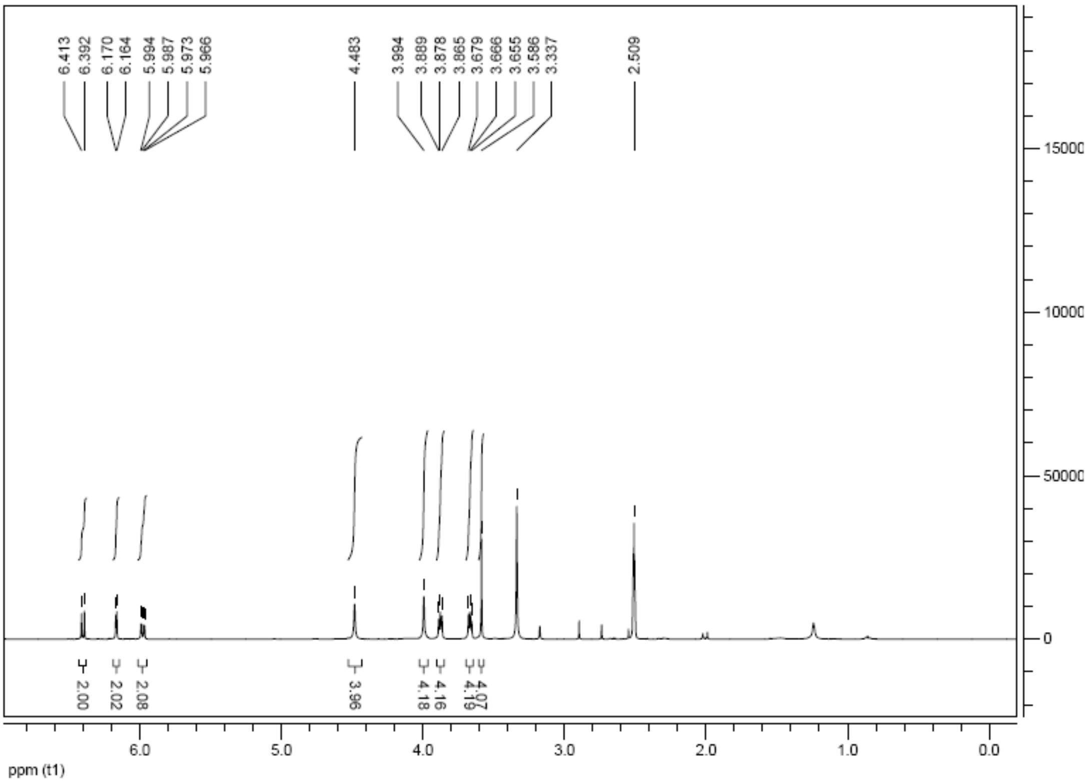

Figure S13. ^{1}H NMR (400 MHz) of compound **6b** in d_6-DMSO.

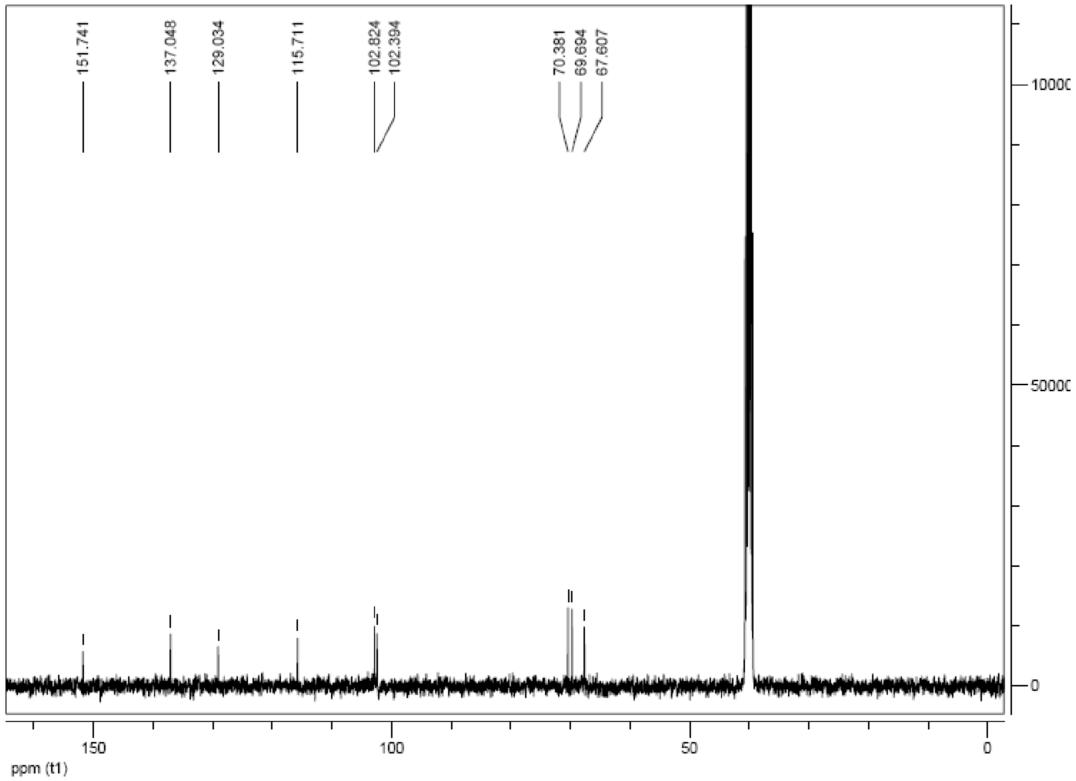

Figure S14. ^{13}C NMR (75.4 MHz) of compound **6b** in d_6-DMSO.

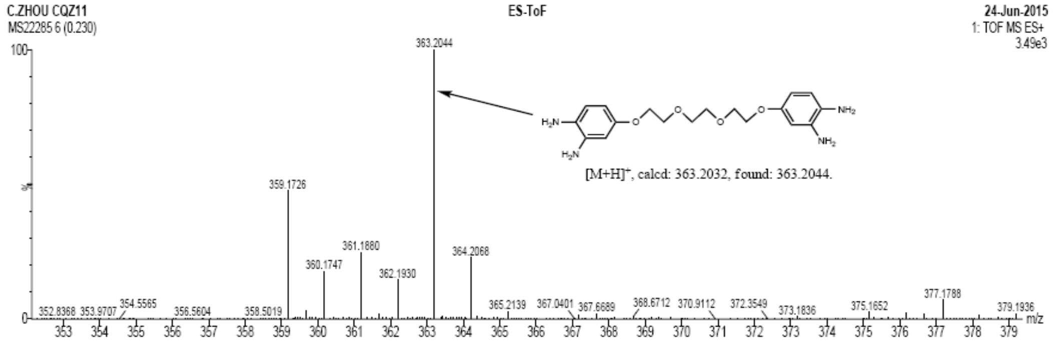

Figure S15. HR-ESI-MS of compound **6b**.

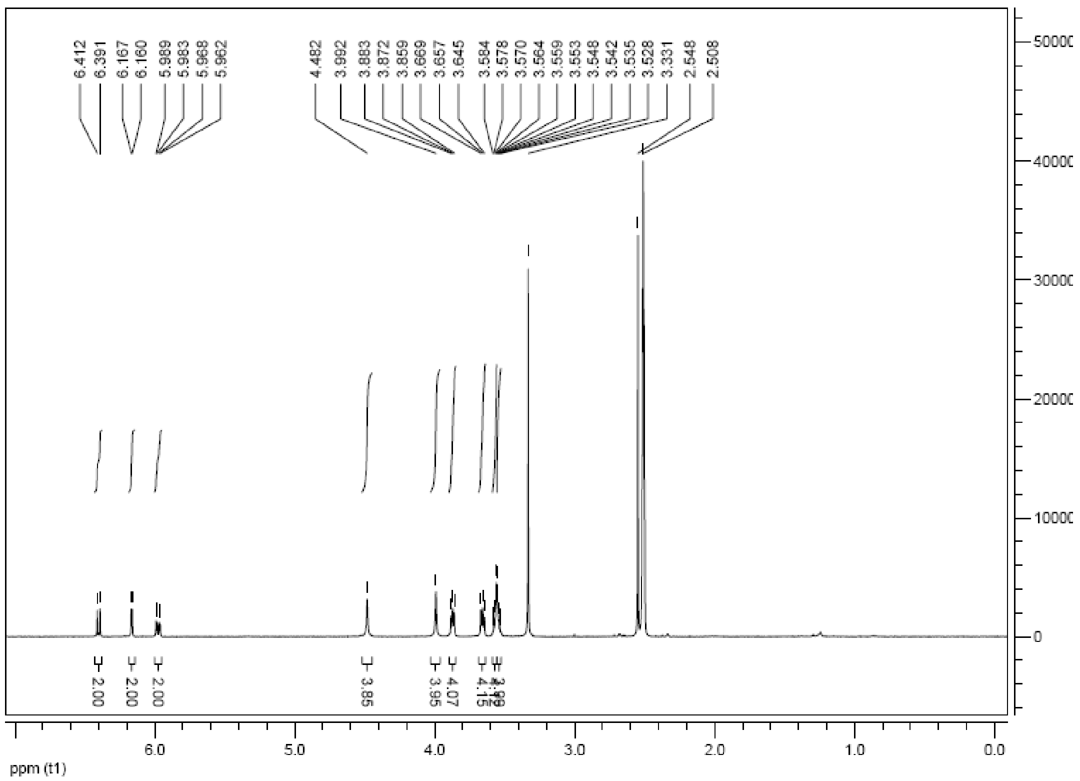

Figure S16. ^{1}H NMR (400 MHz) of compound **6c** in d_6-DMSO.

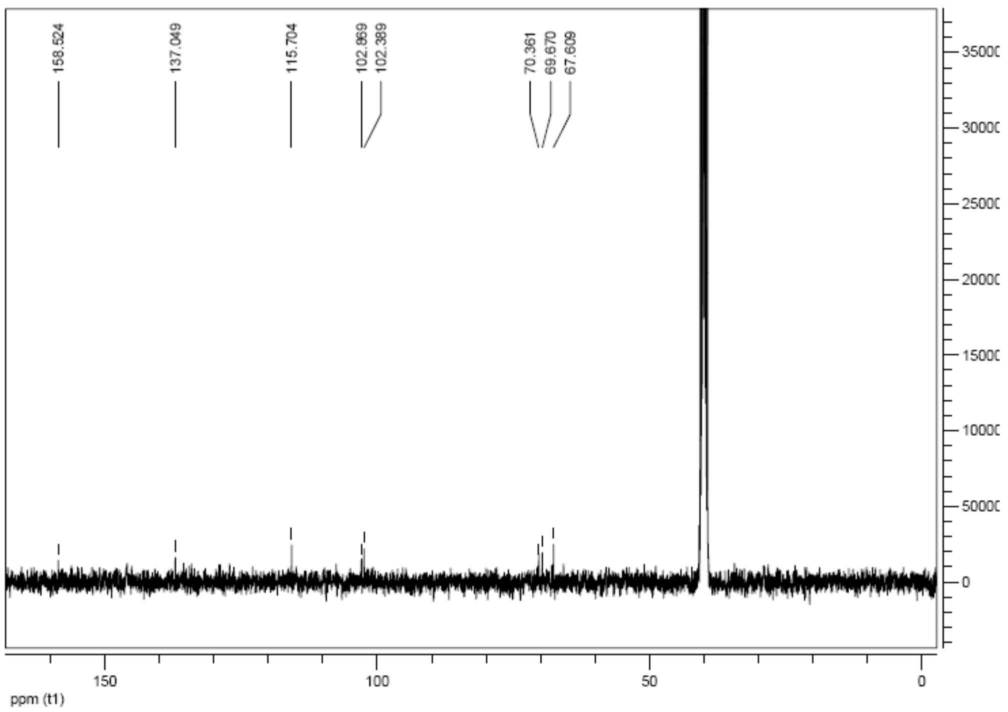

Figure S17. ^{13}C NMR (75.4 MHz) of compound **6c** in d_6-DMSO.

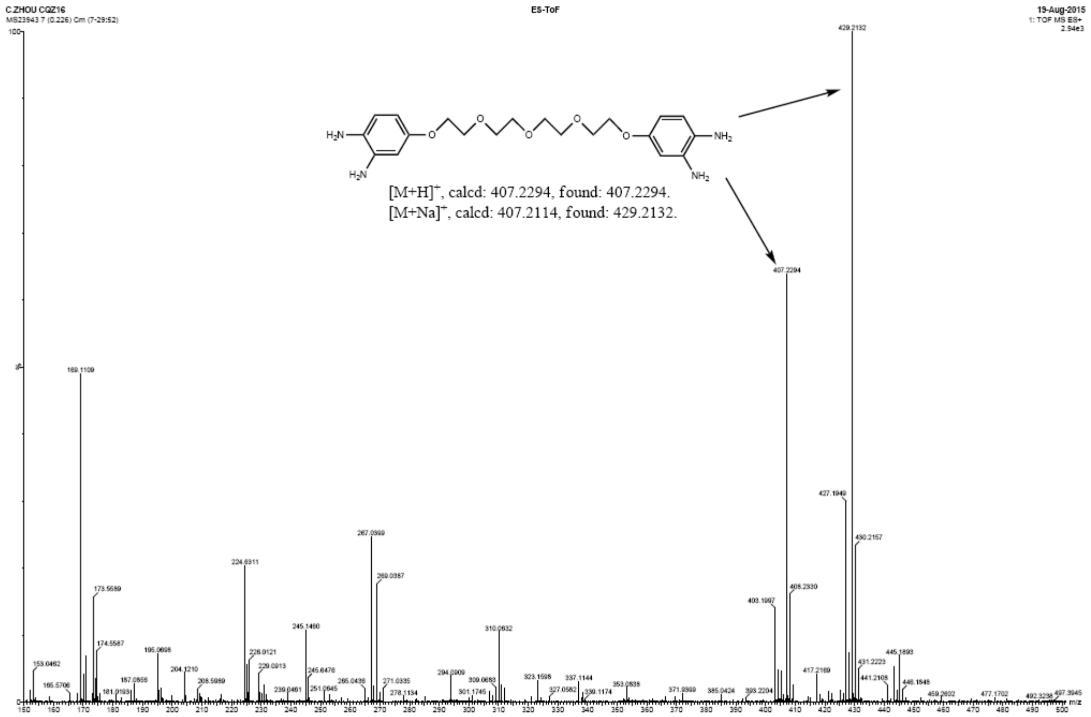

Figure S18. HR-ESI-MS of compound **6c**.

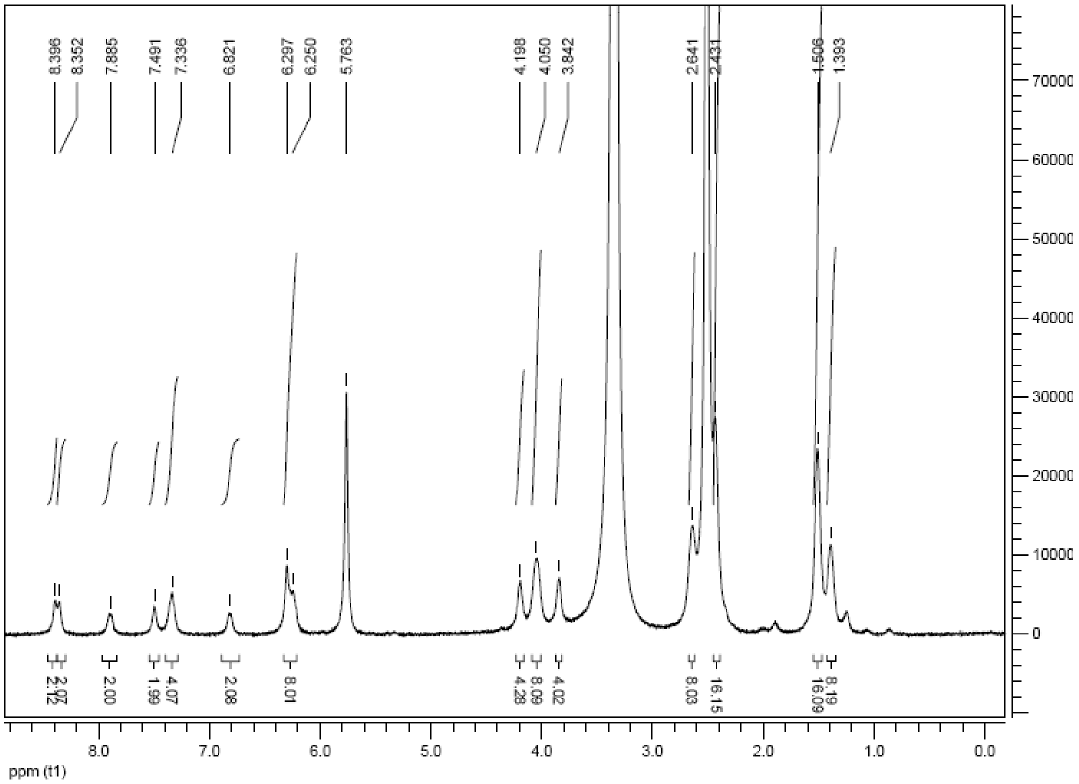

Figure S19. ^{1}H NMR (400 MHz) of complex **2a** in d_6-DMSO.

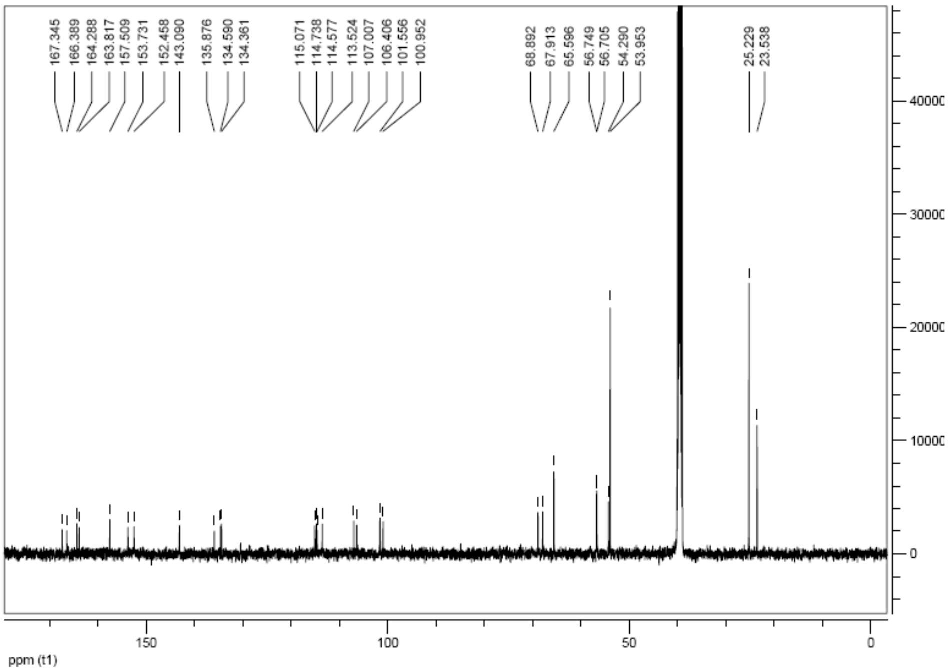

Figure S20. ^{13}C NMR (75.4 MHz) of complex **2a** in d_6-DMSO.

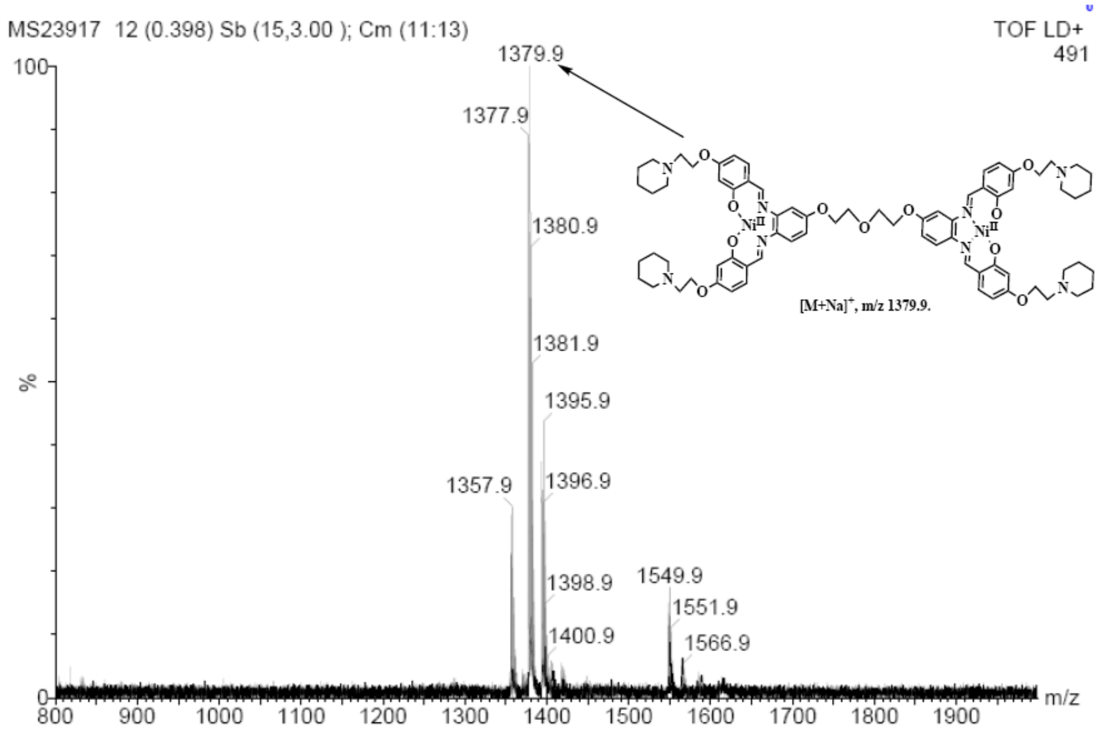

Figure S21. MALDI-TOF MS of complex **2a**.

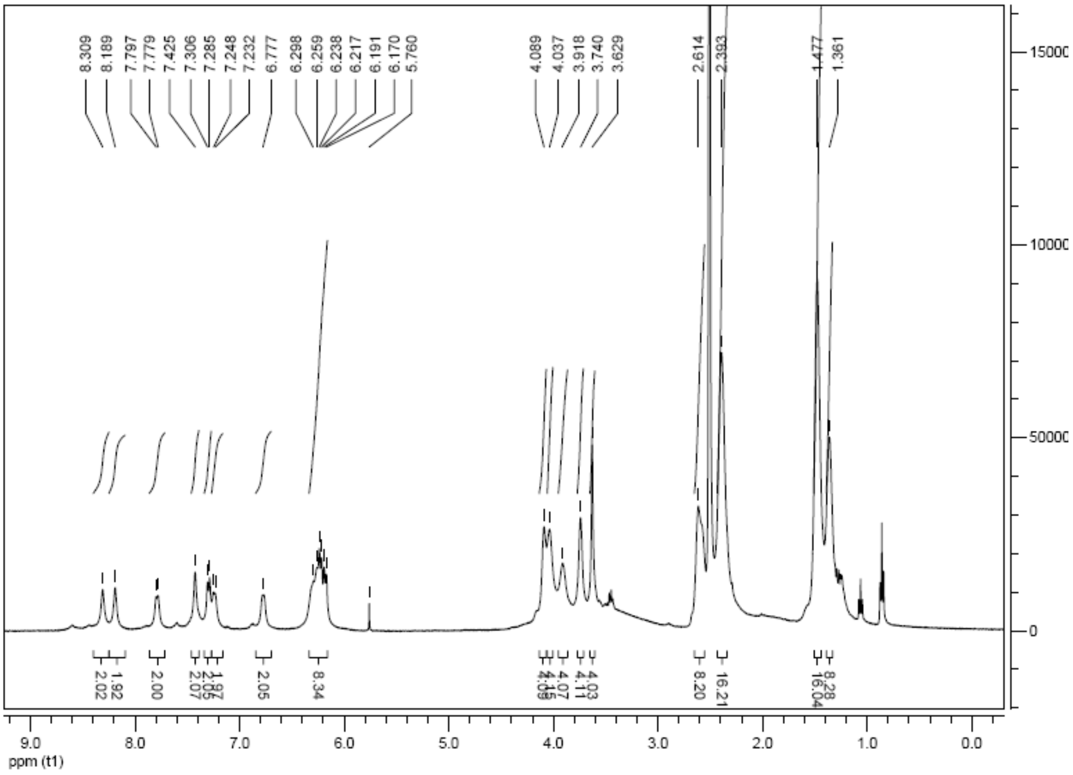

Figure S22. ^{1}H NMR (400 MHz) of complex **2b** in d_6-DMSO.

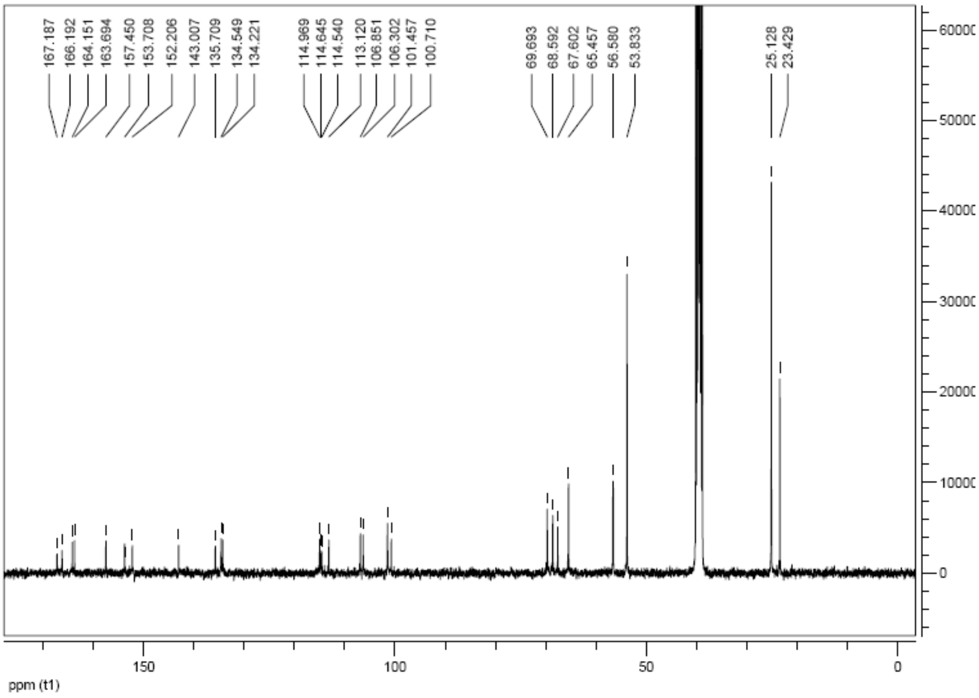

Figure S23. ^{13}C NMR (75.4 MHz) of complex **2b** in d_6-DMSO.

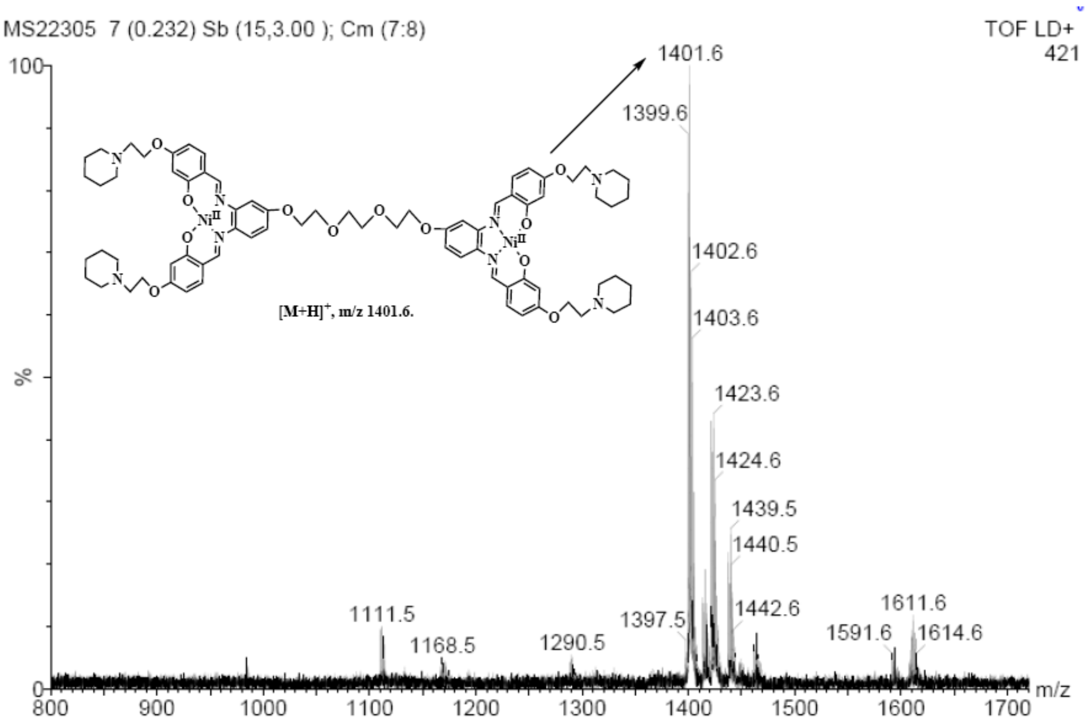

Figure S24. MALDI-TOF MS of complex **2b**.

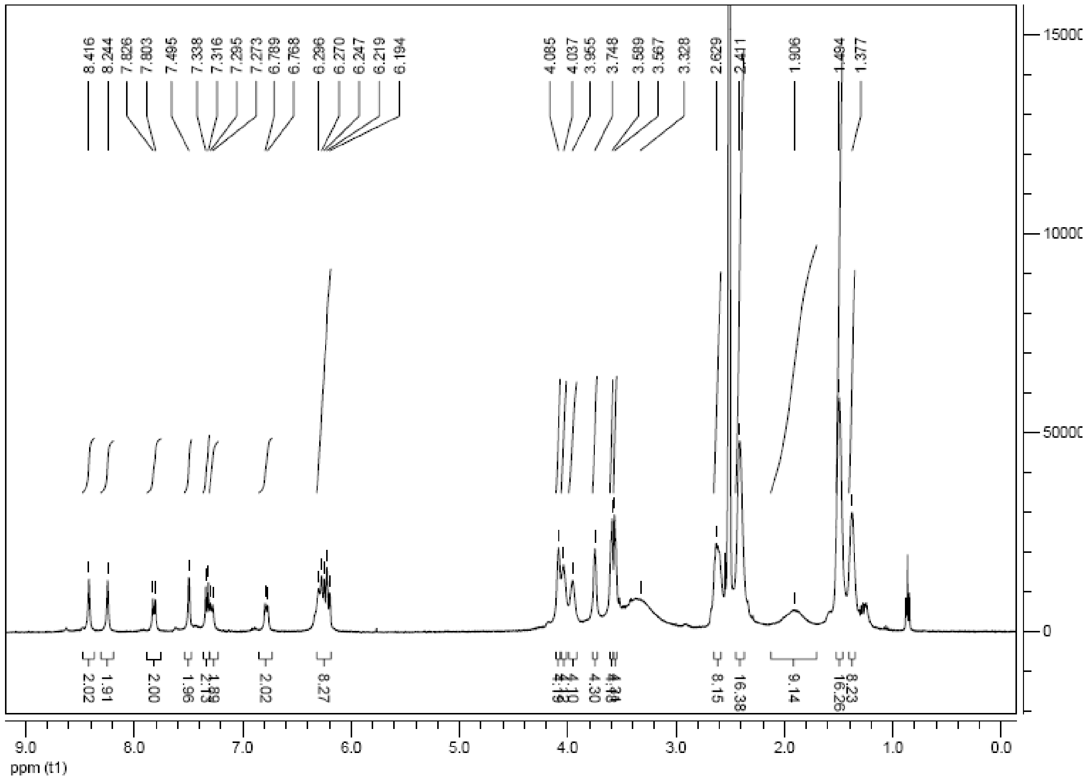

Figure S25. ^{1}H NMR (400 MHz) of complex **2c** in d_6-DMSO.

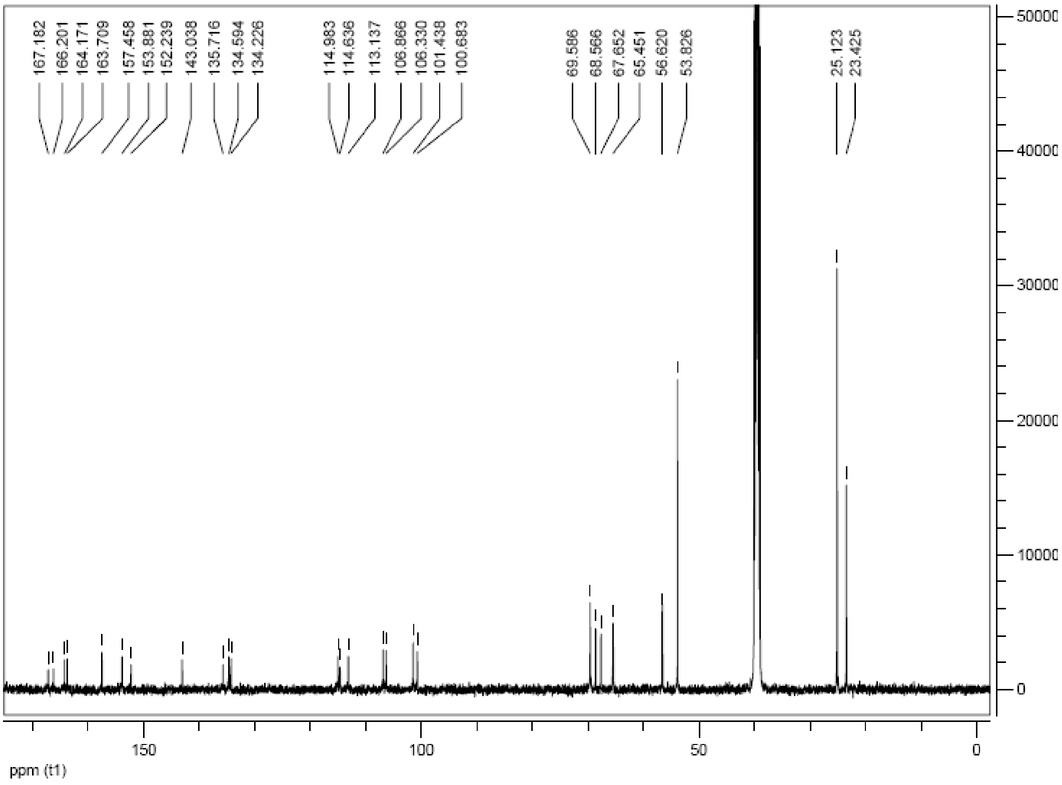

Figure S26. ^{13}C NMR (75.4 MHz) of complex **2c** in d_6-DMSO.

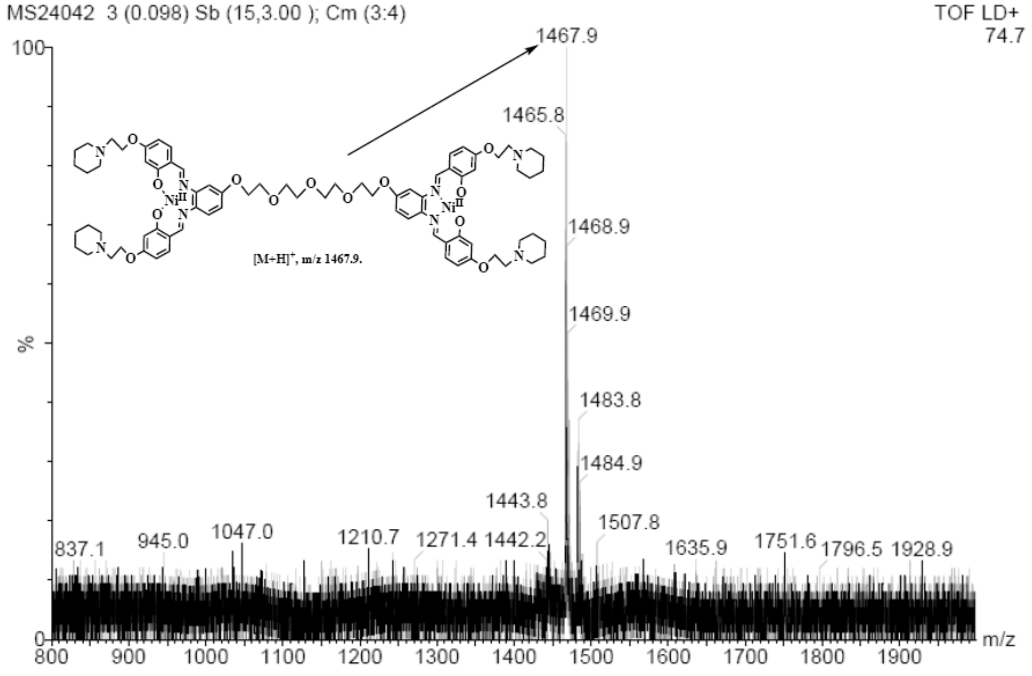

Figure S27. MALDI-TOF MS of complex **2c**.

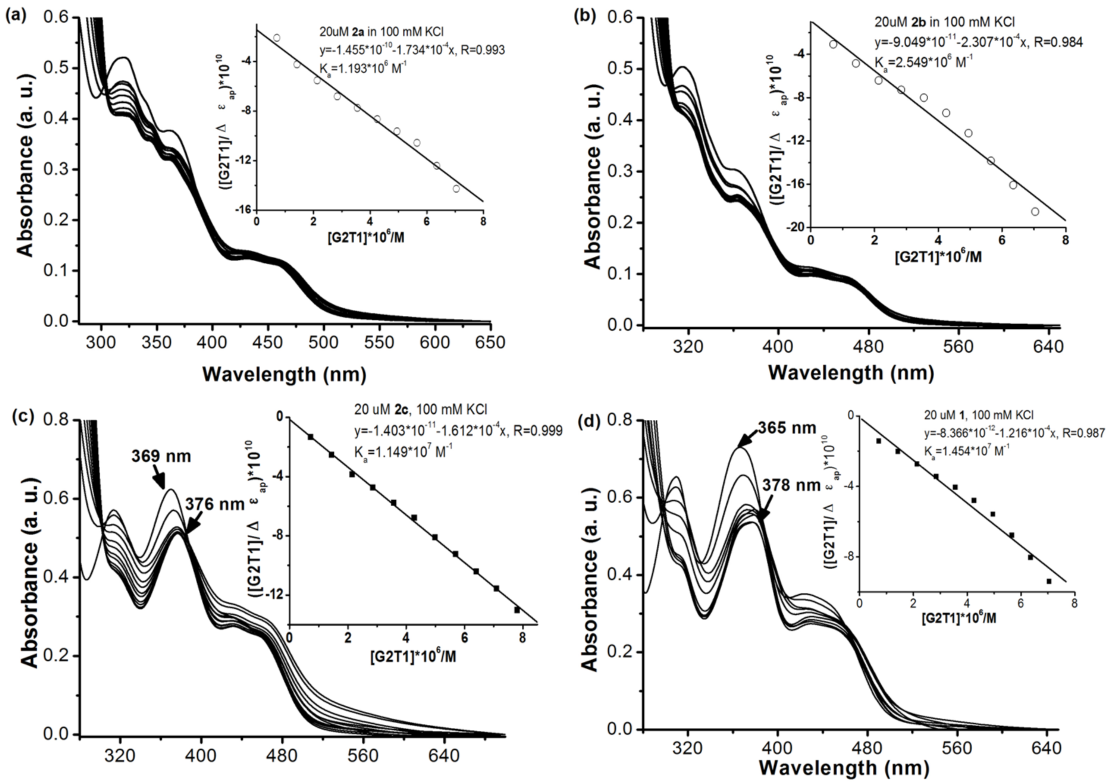

Figure S28. UV-Vis absorption spectra of nickel complexes **2a** (a), **2b** (b), **2c** (c) and **1** (d) ([complex]=20 μM) with increasing the concentration (from 0~10 μM in 10 mM Tris-HCl and 100 mM KCl, pH 7.04) of G2T1 DNA. Inset: a reciprocal plot of ([G2T1]/$\Delta\varepsilon_{ap}$)×10^{10} versus [G2T1]×10^{6}, $\Delta\varepsilon_{ap}=(A_{observed}-A_{free\ complex})$/[complex].

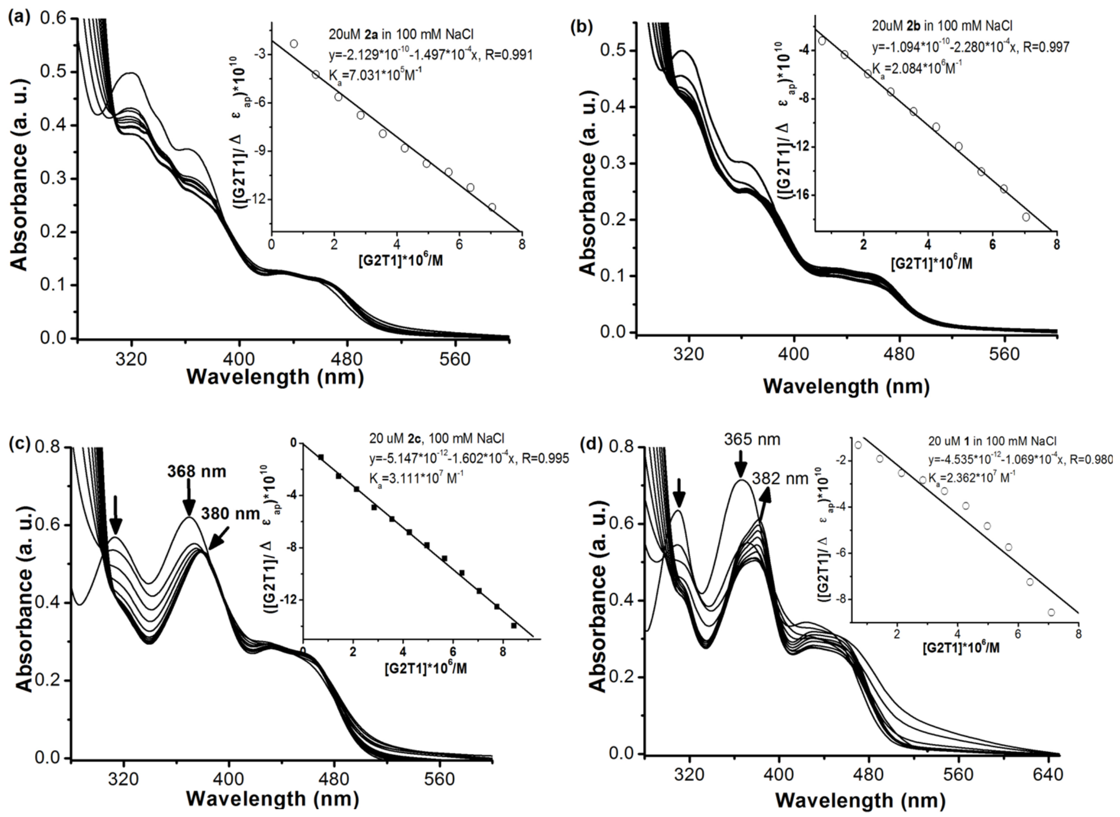

Figure S29. UV-Vis absorption spectra of nickel complexes **2a** (a), **2b** (b), **2c** (c) and **1** (d) ([complex]=20 μM) with increasing the concentration (from 0~10 μM in 10 mM Tris-HCl and 100 mM NaCl, pH 7.04) of G2T1 DNA. Inset: Inset: a reciprocal plot of ([G2T1]/$\Delta\varepsilon_{ap}$)×10^{10} versus [G2T1]×10^6, $\Delta\varepsilon_{ap}$=($A_{observed}$–$A_{free\ complex}$)/[complex].

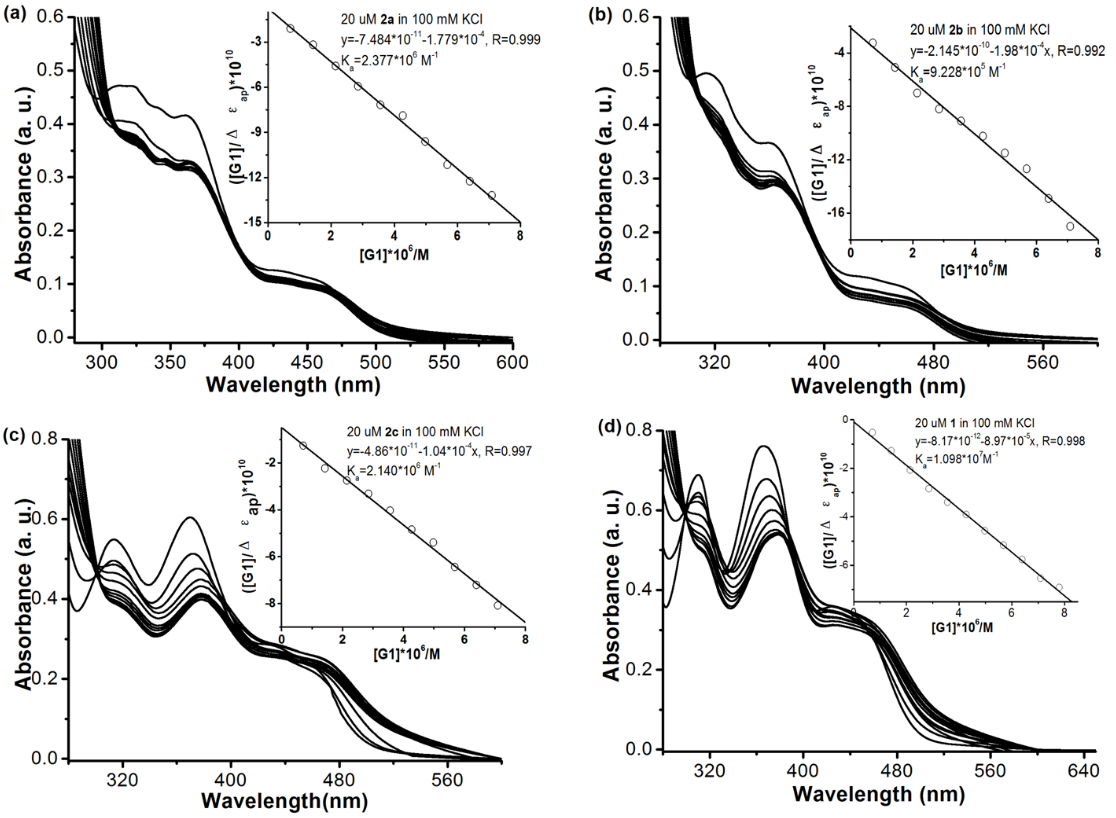

Figure S30. UV-Vis absorption spectra of nickel complexes **2a** (a), **2b** (b), **2c** (c) and **1** (d) ([complex]=20 μM) with increasing the concentration (from 0~10 μM in 10 mM Tris-HCl and 100 mM KCl, pH 7.04) of G1 DNA. Inset: a reciprocal plot of ([G1]/$\Delta\varepsilon_{ap}$)×10^{10} versus [G1]×10^6, $\Delta\varepsilon_{ap}$ = ($A_{observed}$–$A_{free\ complex}$) / [complex].

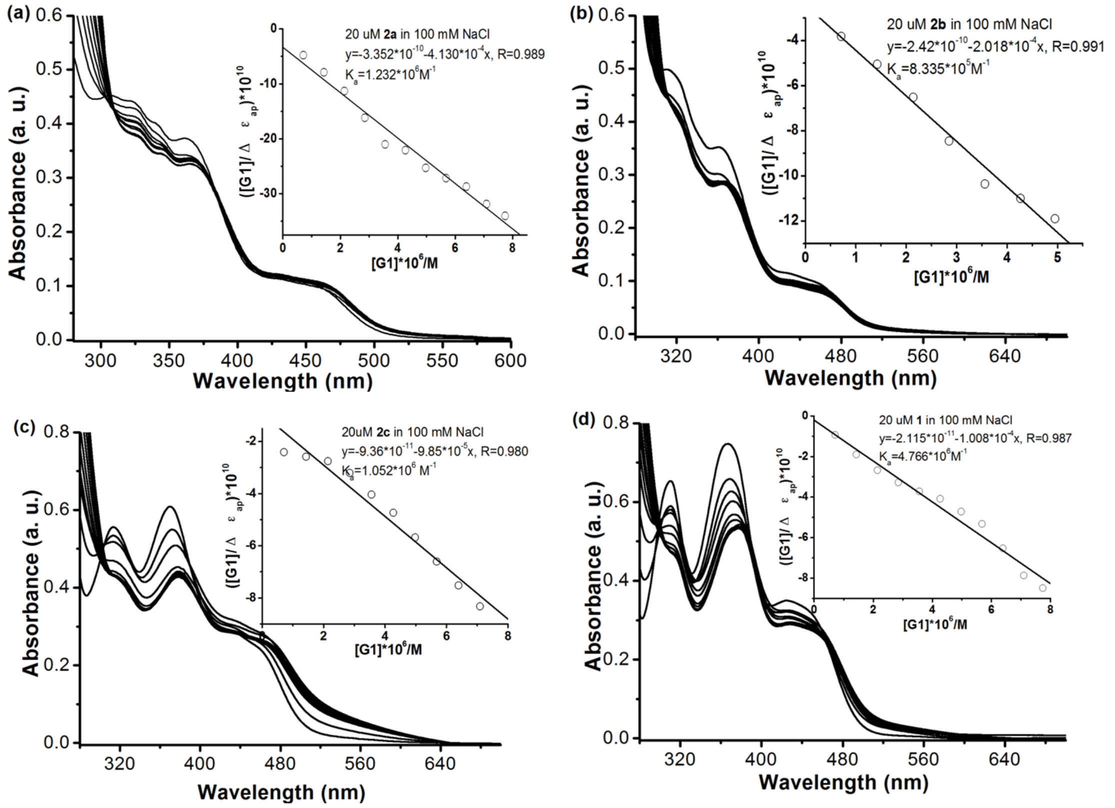

Figure S31. UV-Vis absorption spectra of nickel complexes **2a** (a), **2b** (b), **2c** (c) and **1** (d) ([complex]=20 μM) with increasing the concentration (from 0~10 μM in 10 mM Tris-HCl and 100 mM NaCl, pH 7.04) of G1 DNA. Inset: a reciprocal plot of $([G1]/\Delta\varepsilon_{ap})\times10^{10}$ versus $[G1]\times10^{6}$, $\Delta\varepsilon_{ap}=(A_{observed}-A_{free\ complex})/[complex]$.

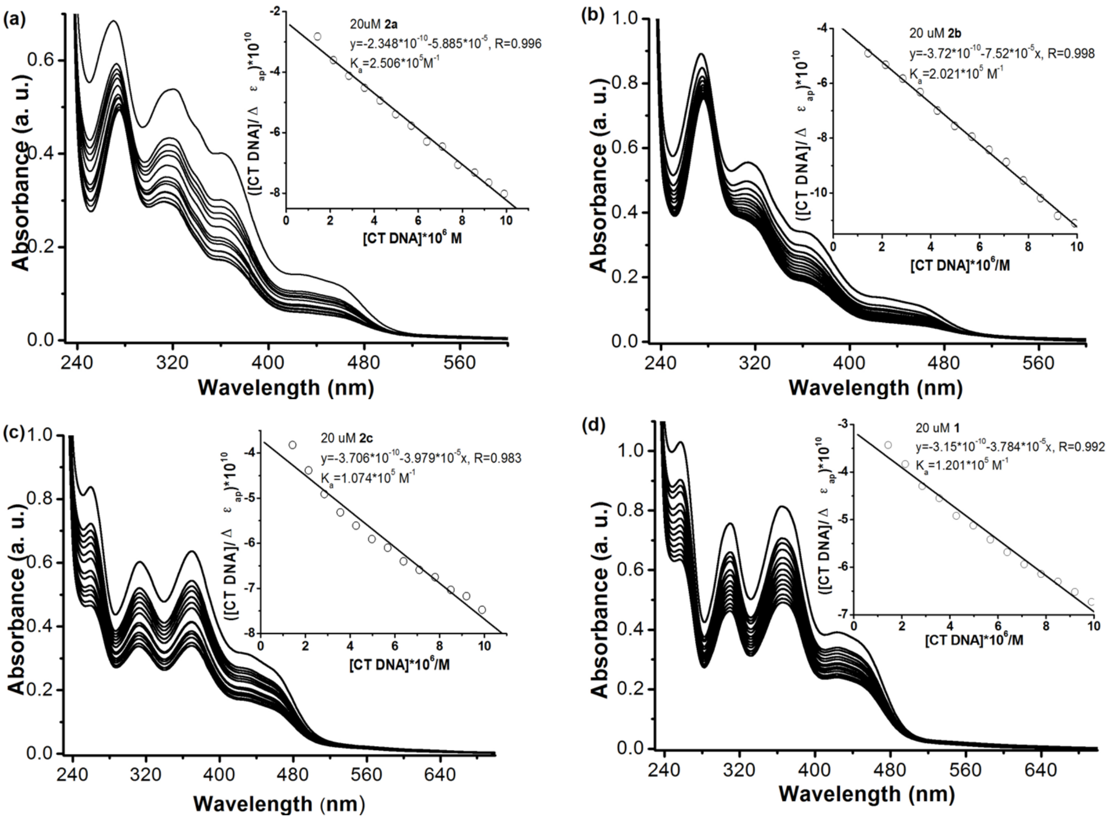

Figure S32. UV-Vis absorption spectra of nickel complexes **2a** (a), **2b** (b), **2c** (c) and **1** (d) ([complex]=20 μM) with increasing the concentration (from 0~10 μM in 10 mM Tris-HCl and 100 mM NaCl, pH 7.04) of CT DNA. Inset: a reciprocal plot of ([CT DNA]/$\Delta\varepsilon_{ap}$)×10^{10} versus [CT DNA] ×10^6, $\Delta\varepsilon_{ap} = (A_{observed}–A_{free\ complex})$/[complex].

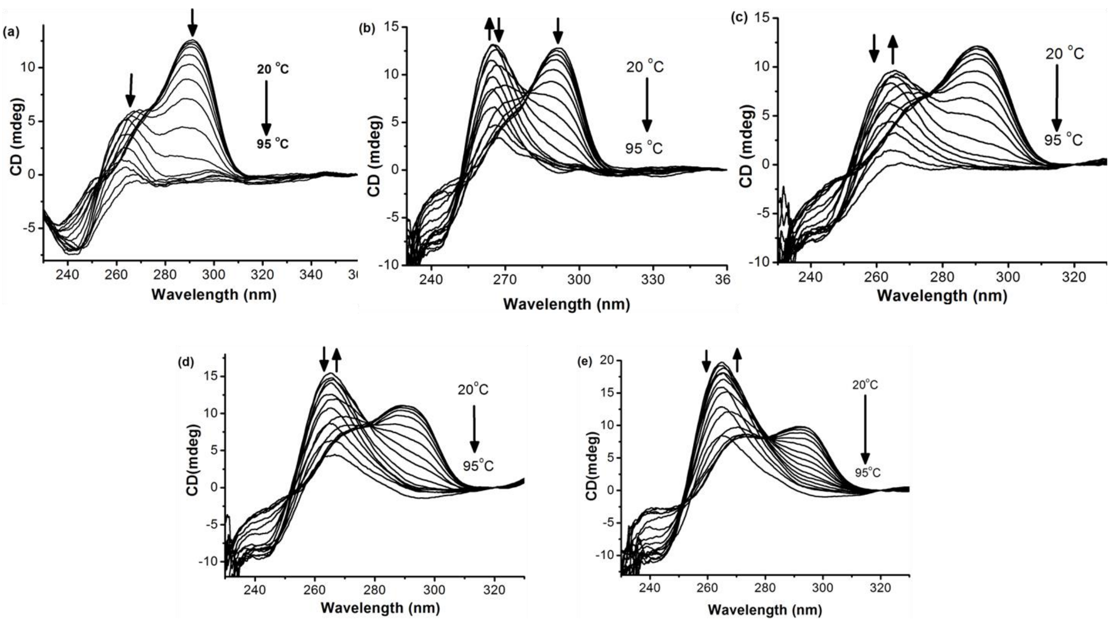

Figure S33. CD spectra of quadruplex dimer (G2T1, 2.5 μM) from 20 °C to 95 °C without (a) and with complexes **2a** (5 μM, b), **2b** (5 μM, c), **2c** (5 μM, d) and **1** (10 μM, e) in the buffer of 10 mM Tris-HCl and 100 mM KCl (pH 7.04).

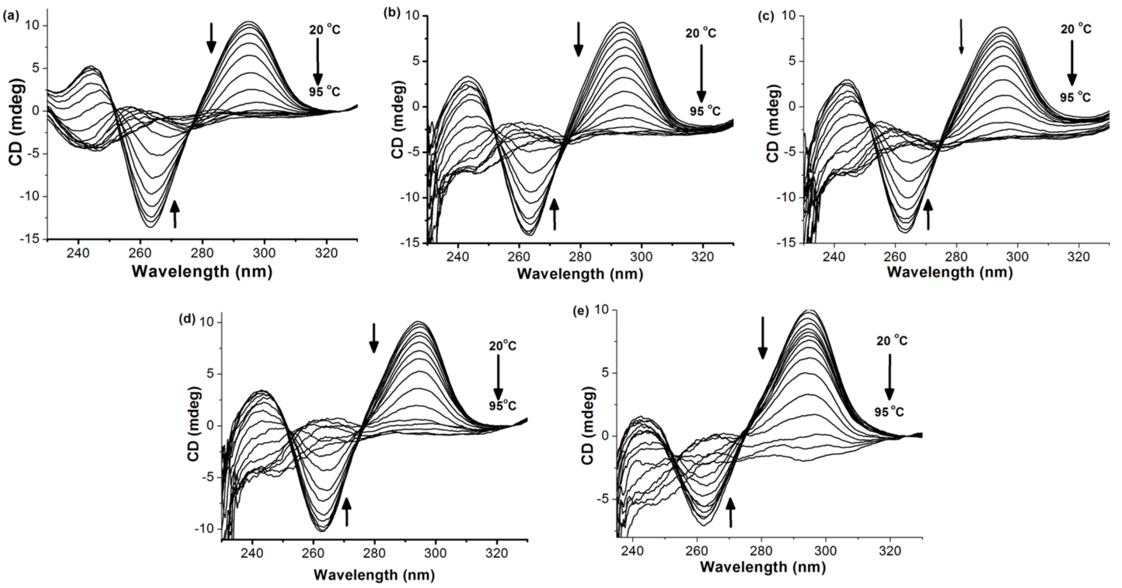

Figure S34. CD spectra of quadruplex dimer (G2T1, 2.5 μM) from 20 °C to 95 °C without (a) and with complexes **2a** (5 μM, b), **2b** (5 μM, c), **2c** (5 μM, d), and **1** (10 μM, e) in the buffer of 10 mM Tris-HCl and 100 mM NaCl (pH 7.04).

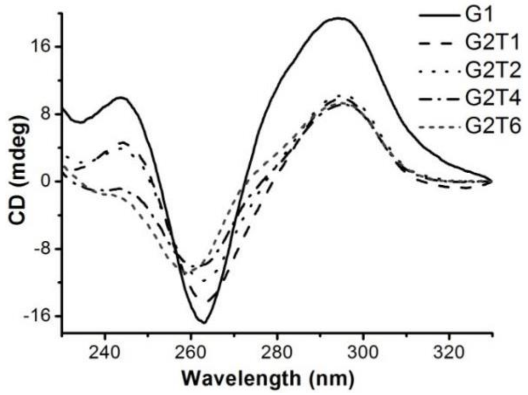

Figure S35. CD spectra of G1 (5 μM), G2T1 (2.5 μM), G2T2 (2.5 μM), G2T4 (2.5 μM) and G2T6 (2.5 μM) in 10 mM Tris-HCl buffer (100 mM NaCl, pH 7.04).

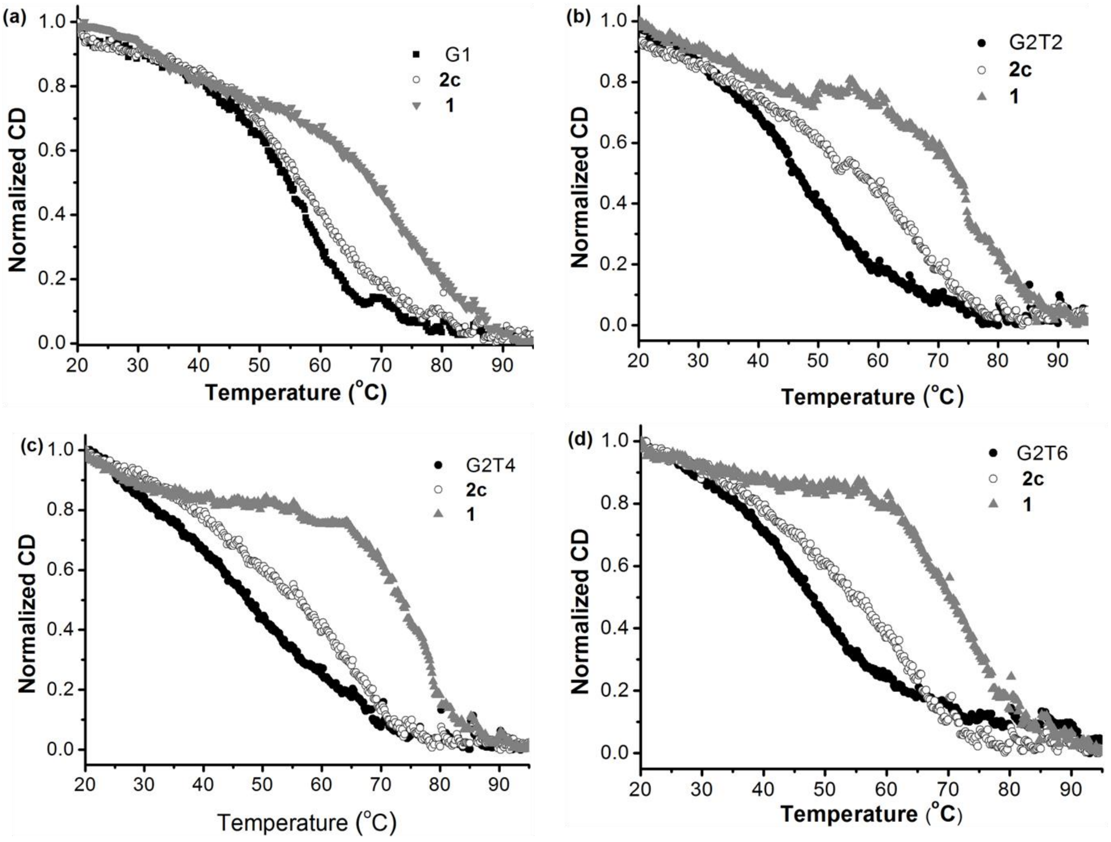

Figure S36. CD melting profiles at 295 nm for monomeric quadruplex G1 (a, 5.0 μM), and a series of dimeric quadruplexes G2T2 (b, 2.5 μM), G2T4 (c, 2.5 μM) and G2T6 (d, 2.5 μM), respectively, when bound to di-nickel complex **2c** (5.0 μM) and mono nickel complex **1** (10.0 μM) in 10 mM Tris-HCl and 100 mM NaCl (pH 7.04).

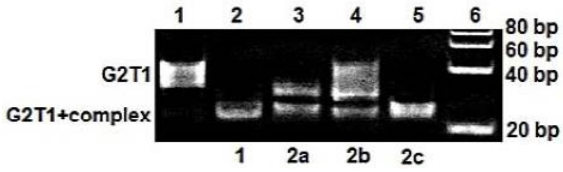

Figure S37. GE analysis of G2T1 in 10 mM Tris-HCl and 100 mM NaCl (pH 7.04) in the presence of four nickel complexes: lane 1, G2T1 (8 μM); lane 2~5, G2T1 (8 μM) with complex **1** (32 μM), **2a** (16 μM), **2b** (16 μM) and **2c** (16 μM), respectively; lane 6, DNA ladder.

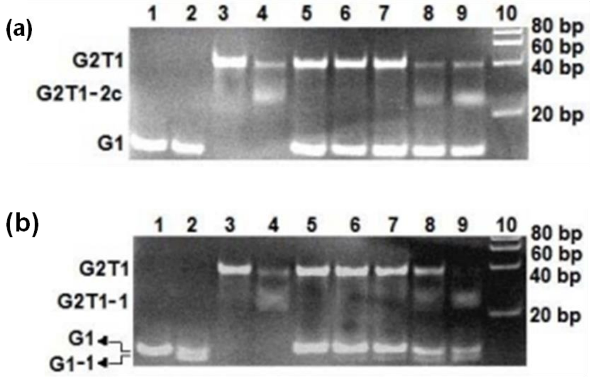

Figure S38. (a) GE analysis of G2T1 in 10 mM Tris-HCl and 100 mM KCl (pH 7.04) in the presence of **2c**. Lanes 1~2: G1 (16 μM) in the absence and presence of complex **2c** (16 μM); lanes 3~4: G2T1 (8 μM) in the absence and presence of complex **2c** (16 μM); lane 5: a mixture of G1 (16 μM) and G2T1 (8 μM); lanes 6~9: mixtures of G1 (16 μM) and G2T1 (8 μM) in the presence of 4, 8, 16 and 32 μM of complex **2c**, respectively; lane 10: DNA ladder. (b) GE analysis of G1, G2T1 and their mixture in 10 mM Tris-HCl and 100 mM KCl (pH 7.04) in the presence of complex **1**. Lanes 1~2: G1 (16 μM) in the absence and presence of complex **1** (32 μM); lanes 3~4: G2T1 (8 μM) in the absence and presence of complex **1** (32 μM); lane 5: a mixture of G1 (16 μM) and G2T1 (8 μM); lanes 6~9: mixtures of G1 (16 μM) and G2T1 (8 μM) in the presence of 8, 16, 32 and 64 μM of complex **1**, respectively; lane 10: DNA ladder.
